# Tfap2a and 2b act downstream of Ptf1a to promote amacrine cell differentiation during retinogenesis

**DOI:** 10.1186/s13041-015-0118-x

**Published:** 2015-05-13

**Authors:** Kangxin Jin, Haisong Jiang, Dongchang Xiao, Min Zou, Jun Zhu, Mengqing Xiang

**Affiliations:** State Key Laboratory of Ophthalmology, Zhongshan Ophthalmic Center, Sun Yat-sen University, 54 South Xianlie Road, Guangzhou, 510060 China; Center for Advanced Biotechnology and Medicine and Department of Pediatrics, Rutgers University-Robert Wood Johnson Medical School, 679 Hoes Lane West, Piscataway, NJ 08854 USA; Systems Biology Center, National Heart, Lung and Blood Institute, National Institutes of Health, Bethesda, MD 20892 USA; Present address: Institute for Cell Engineering, Departments of Neurology and Neuroscience, Johns Hopkins University School of Medicine, 733 North Broadway, Baltimore, MD 21206 USA

**Keywords:** Tfap2, Ptf1a, Foxn4, Amacrine cell, Horizontal cell, Retinal development

## Abstract

Retinogenesis is a precisely controlled developmental process during which different types of neurons and glial cells are generated under the influence of intrinsic and extrinsic factors. Three transcription factors, Foxn4, ROR*β*1 and their downstream effector Ptf1a, have been shown to be indispensable intrinsic regulators for the differentiation of amacrine and horizontal cells. At present, however, it is unclear how Ptf1a specifies these two cell fates from competent retinal precursors. Here, through combined bioinformatic, molecular and genetic approaches in mouse retinas, we identify the Tfap2a and Tfap2b transcription factors as two major downstream effectors of Ptf1a. RNA-seq and immunolabeling analyses show that the expression of Tfap2a and 2b transcripts and proteins is dramatically downregulated in the *Ptf1a* null mutant retina. Their overexpression is capable of promoting the differentiation of glycinergic and GABAergic amacrine cells at the expense of photoreceptors much as misexpressed Ptf1a is, whereas their simultaneous knockdown has the opposite effect. Given the demonstrated requirement for Tfap2a and 2b in horizontal cell differentiation, our study thus defines a Foxn4/RORβ1-Ptf1a-Tfap2a/2b transcriptional regulatory cascade that underlies the competence, specification and differentiation of amacrine and horizontal cells during retinal development.

**Electronic supplementary material:**

The online version of this article (doi:10.1186/s13041-015-0118-x) contains supplementary material, which is available to authorized users.

## Introduction

In the mammalian retina, there are six major types of neurons, including ganglion, amacrine, horizontal, bipolar, cone and rod cells. During retinogenesis, the generation of proper quantity and types of these neurons in the correct position at the right time is essential for the assembly of a fully functional retina. This developmental process is primarily controlled by intrinsic programs coded largely by transcription factors, as well as influenced by various extrinsic factors such as hormones, cytokines, chemokines, cell-cell interactions, and so on [1-4].

The amacrine, horizontal and bipolar cells in the retina are interneurons that serve to relay, integrate and modulate visual signals from photoreceptors to ganglion cells. The amacrine cells, which modulate synaptic activity between bipolar and ganglion cells, are the most diverse cell type within the retina [5,6]. In mammals, they can be classified into at least 28 different subtypes based on criteria such as morphology, sublaminar location, and neurotransmitter types (e.g. glycinergic, GABAergic, dopaminergic or cholinergic) [7,8]. Glycinergic amacrine cells are usually small-field neurons with diffuse dendritic trees [9,10], whereas GABAergic amacrine cells generally have wider dendritic fields than those of glycinergic cells [11,12]. Horizontal cells constitute a class of retinal interneurons that modulate signal transmission between photoreceptors and bipolar cells.

Although a few intrinsic and extrinsic factors have been identified that affect amacrine and horizontal cell development, the genetic regulatory network that controls their determination and differentiation remains to be defined. Ablation of *Foxn4* in mice causes a loss of all horizontal cells and the great majority of amacrine cells. Conversely, its misexpression in mouse and chick retinas promoted the amacrine and horizontal cell fates [13,14]. Gene expression profiling identified *Ptf1a* as one of the most downregulated genes in *Foxn4* null mutant retinas, and in *Ptf1a* mutants, there is similar loss of all horizontal cells and the majority of amacrine cells; however, there is no change in *Foxn4* expression [15,16], thereby defining a Foxn4-Ptf1a pathway controlling the specification of amacrine and horizontal cells [4,15,17]. Indeed, Ptf1a overexpression has been shown to promote amacrine and horizontal cell differentiation in the chick, *Xenopus* and zebrafish [18-20]. This pathway has been expanded recently to include the retinoid-related orphan receptor isoform β1 (RORβ1), whose inactivation phenocopies the *Foxn4* and *Ptf1a* mutants in amacrine and horizontal cell development and downregulates the expression of *Ptf1a* but not *Foxn4* [21]. It seems that RORβ1 acts in parallel with Foxn4 to activate *Ptf1a* expression [21]. At present, it is unclear what are the Ptf1a downstream effectors that mediate its function during retinal cell development.

We provide evidence in this study that Tfap2a and Tfap2b are positioned downstream of Ptf1a in the transcription factor pathway governing amacrine and horizontal cell development. These two factors belong to the Activating Enhancer Binding Protein 2 family, for which currently at least five members (2a/α, 2b/β, 2c/γ, 2d/δ, 2e/ε) have been identified. Tfap2a and 2b recognize and bind to the consensus sequence 5'-GCCNNNGGC-3' and activate genes involved in a large spectrum of important biological functions including eye, neural tube, ear, kidney, and limb development [22,23]. Mutations in human *TFAP2A* are associated with the Branchio-Oculo-Facial Syndrome [24,25]. In the early retina, both Tfap2a and 2b are expressed in the developing amacrine and horizontal cells and conditional ablation of *Tfap2a* alone is insufficient to cause any defect in either cell population [26-28]. However, a double mutant lost all of the horizontal cells but displayed no obvious change in the number of amacrine cells except for a minor migratory defect [28], suggesting that Tfap2a and 2b are redundantly required for horizontal cell differentiation but may be nonessential for amacrine cell differentiation. Here, however, we provide RNA-seq evidence to position Tfap2a and 2b downstream of Ptf1a, and demonstrate that they can mediate the crucial function of Ptf1a in amacrine cell development, using both gain- and loss-of-function approaches.

## Results

### Tfap2a and 2b are genetically downstream of the Foxn4-Ptf1a pathway

**Figure 1:**
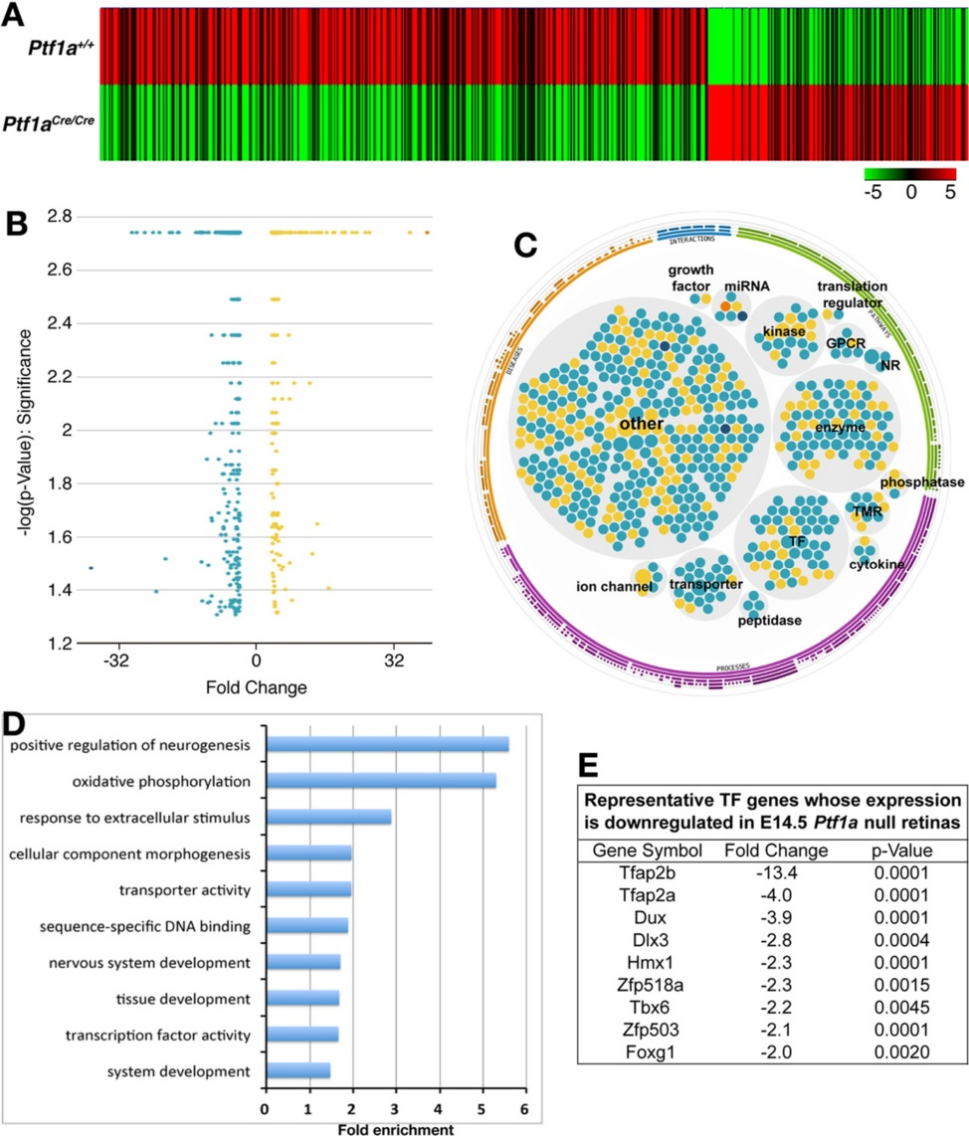
RNA-seq analysis of differentially expressed genes in E14.5 *Ptf1a* ^*Cre/Cre*^ retinas. **(A)** Cluster analysis reveals a large group of significantly down-regulated genes and a smaller group of significantly upregulated genes in the mutant retina. **(B)** Volcano plot (significance vs fold change) of significantly altered genes (fold change ≥ 2 and p < 0.05). **(C)** Differentially expressed genes grouped by molecular function. Cyan indicates downregulated genes and yellow/orange upregulated genes. GPCR, G-protein coupled receptor; NR, ligand-dependent nuclear receptor; TF, transcription factor; TMR, transmembrane receptor. **(D)** Representative functional GO terms significantly enriched for the differentially expressed genes. **(E)** Representative transcription factor genes whose expression is significantly altered in the mutant retina.

To explore the molecular basis by which Ptf1a controls amacrine and horizontal cell development, we carried out RNA-seq analysis to identify genes differentially expressed in *Ptf1a* mutant retinas. RNA was extracted from *Ptf1a*^*+*/*+*^ and *Ptf1a*^*Cre/Cre*^ retinas at E14.5 when amacrine and horizontal cells are being born and Ptf1a function is required. This analysis yielded 224 genes whose expression level is downregulated or upregulated by 2-fold or more in the mutant retina (Figure 1A, B; Additional file 1: Table S1). These include genes encoding transcription factors, G-protein coupled receptors, kinases and transporters, etc. (Figure 1C). Consistent with the crucial role of Ptf1a in retinal development, we found that the differentially expressed genes are enriched with GO (Gene Ontology) terms such as positive regulation of neurogenesis, nervous system development, tissue development, cellular component morphogenesis, response to extracellular stimulus, transcription factor activity, and so on (Figure 1D).

**Figure 2:**
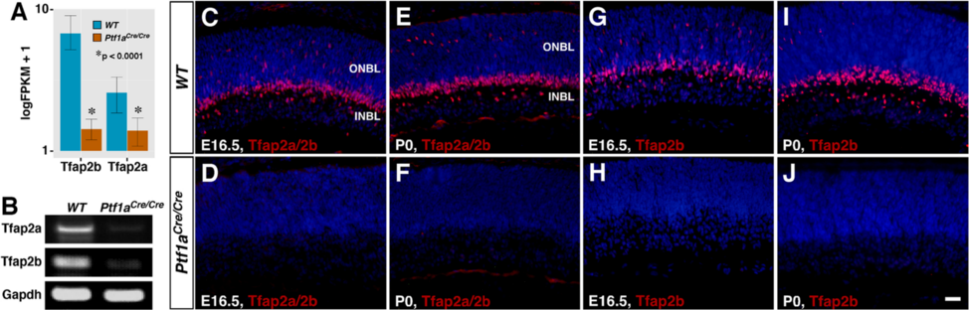
Downregulation of Tfap2a and 2b expression in *Ptf1a* mutant retinas. **(A)** Expression plot shows a dramatic reduction of *Tfap2a* and *2b* expression in the *Ptf1a* ^*Cre/Cre*^ retina compared to the wild type (WT), as measured by FPKM. **(B)** Semiquantitative RT-PCR assay reveals a great decrease of *Tfap2a* and *2b* RNA levels in the null retina. *Gapdh* was used as an internal control to confirm that equal amount of cDNA was used for each sample. **(C-J)** Sections from E16.5 and P0 retinas of *Ptf1a* ^*+/+*^ and *Ptf1a* ^*cre/cre*^ mice were immunostained with antibodies that react with both Tfap2a and 2b **(C-F)** or only Tfap2b **(G-J)**, and weakly counterstained with DAPI. There is almost a complete loss of Tfap2-immunoreactive cells in *Ptf1a* ^*cre/cre*^ retinas. Abbreviations: INBL, inner neuroblastic layer; ONBL, outer neuroblastic layer. Scale bar: C-J, 25 μm.

Among the genes differentially expressed in *Ptf1a* null mutant retinas, transcription factor genes constitute one of the largest functional groups (Figure 1C). These include *Tfap2a* and *Tfap2b*, which are downregulated by 4- and 13-fold, respectively (Figure 1E; Figure 2A). Similarly, our previous microarray data show that these two genes are downregulated by 4- and 9-fold, respectively, in the E14.5 *Foxn4* null retina [29]. To confirm the RNA-seq data, we measured RNA levels of these two genes in E14.5 wild type and *Ptf1a*^*Cre/Cre*^ retinas by semi-quantitative RT-PCR, and found that there was a dramatic decrease in *Tfap2a* and *2b* transcripts in the null retina compared to the control (Figure 2B). In addition, we examined Tfap2a and 2b protein expression levels by immunofluorescence using two antibodies, one of which cross-reacts with both proteins and the other is specific to Tfap2b. Either antibody barely detected any Tfap2a/2b-expressing cells in E16.5 and P0 *Ptf1a* null retinas despite plenty of them present in the control retina (Figure 2C-J).

During mouse retinal development, Tfap2b expression is barely detectable in E12.5 retinas but found in cells scattered within the central region of E13.5 retinas (Additional file 2: Figure S1A, B). From E14.5 to early postnatal stages, Tfap2b-expressing cells gradually concentrate into the presumptive inner nuclear layer (INL) (Additional file 2: Figure S1C, D; Figure 2G, I). In late postnatal and mature retinas, Tfap2b is expressed in numerous amacrine cells located within the inner half of the INL, in all horizontal cells residing at the outer plexiform layer, as well as in a subset of cells in the ganglion cell layer (GCL) (Additional file 2: Figure S1F). Moreover, Tfap2a and 2b are colocalized in most of these cells although the expression of Tfap2a is rather weak in horizontal cells (Additional file 2: Figure S1E-G). A similar spatiotemporal expression pattern was previously reported for Tfap2a during mouse retinal development [26]. However, unlike Tfap2a and 2b, our previous study indicates that Ptf1a expression is limited to retinal precursor cells as its expression is transient, present only in the outer neuroblastic layer throughout development, and has an onset time of E12.5 [15]. These results combined with the RNA-seq data thus suggest that Tfap2a and 2b may function genetically downstream of the Foxn4-Ptf1a pathway and have a role in amacrine and horizontal cell development.

### Ptf1a induces Tfap2b expression and promotes the amacrine and horizontal cell fates

**Figure 3:**
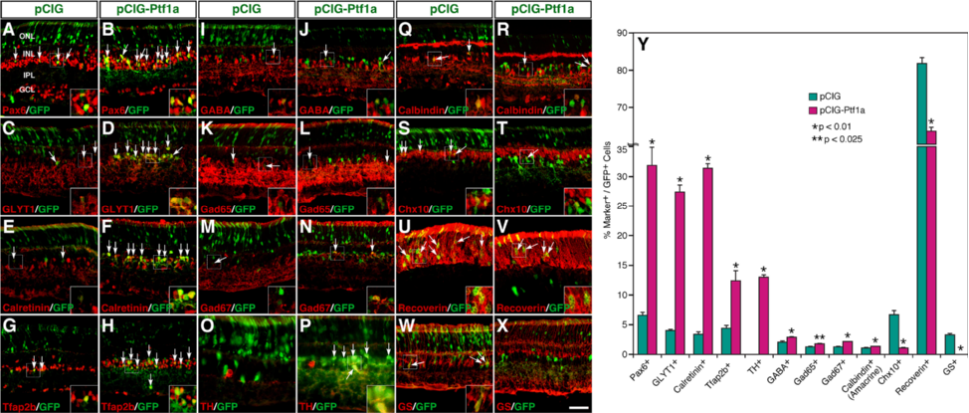
Effect of misexpressed Ptf1a on the formation of different retinal cell types. **(A-X)** Sections from retinas electroporated with pCIG or pCIG-Ptf1a DNA were double-immunostained with an anti-GFP antibody and antibodies against the indicated cell type-specific markers. Misexpressed Ptf1a resulted in overt increase in the number of amacrine cells immunoreactive for Pax6, GLYT1, calretinin, Tfap2b or TH **(A-H, O, P)**, and smaller increase in the number of amacrine cells immunoreactive for GABA, Gad67, Gad65, or calbindin **(I-N, Q, R)**. However, it suppressed the formation of bipolar cells immunoreactive for Chx10 **(S, T)**, photoreceptor cells immunoreactive for recoverin **(U, V)**, and Müller cells immunoreactive for GS **(W, X)**. Arrows point to representative colocalized cells and insets show corresponding outlined regions at a higher magnification. **(Y)** Quantitation of GFP+ cells that become immunoreactive for a series of cell type-specific markers. Each histogram represents the mean ± SD for three retinas. More than 500 GFP+ cells were scored in each retina. Abbreviations: GCL, ganglion cell layer; INL, inner nuclear layer; IPL, inner plexiform layer; ONL, outer nuclear layer. Scale bar: A-N, Q-T, W, X, 39.7 μm; O, P, 30.2 μm; U, V, 23.8 μm.

The drastic downregulation of *Tfap2a* and *2b* expression in *Ptf1a* null retinas suggest that Ptf1a may act upstream of these two genes to activate their expression. We tested this possibility by overexpressing Ptf1a in the mouse retina using the pCIG expression vector carrying a GFP reporter [30-32]. pCIG-Ptf1a and pCIG plasmid DNA (Additional file 3: Figure S2A) was injected into the subretinal space of newborn mice and electroporated into the retina. At P12, we found that forced Ptf1a expression induced 2-fold more Tfap2b + cells in retinas transfected with the pCIG-Ptf1a plasmid than in the control retina (Figure 3G, H, Y). Given that Ptf1a is able to induce Tfap2a expression in the chick retina [18], it appears that the expression of both *Tfap2a* and *2b* may be under positive regulation by Ptf1a.

We further analyzed the laminar position and morphology of GFP+ cells in transfected retinas at P12. In retinas transfected with pCIG-Ptf1a DNA, the fraction of GFP+ cells differentiated as photoreceptors in the ONL (outer nuclear layer) dropped from 81.8% in the control retina to 63.5% (Additional file 3: Figure S2B-D). The percentage of GFP+ cells located in the outer half of the INL (inner nuclear layer) also decreased from 10.0% in the control to 1.3% (Additional file 3: Figure S2B-D). In contrast, the proportion of GFP+ cells distributed within the inner half of the INL dramatically increased from 8.3% in the control to 35.2% (Additional file 3: Figure S2B-D). Thus, Ptf1a misexpression substantially changes the proportions of progeny distributed in different retinal cell layers.

The increased GFP+ cells in the INL of retinas transfected with the pCIG-Ptf1a plasmid displayed an amacrine cell morphology (Additional file 3: Figure S2C). Indeed, we found that ectopically expressed Ptf1a obviously increased the number of GFP+ cells immunoreactive for Pax6, GLYT1, calretinin, or TH (tyrosine hydroxylase), all proteins expressed in amacrine cells (Figure 3A-F, O, P). Quantification of colocalized cells revealed that forced Ptf1a expression dramatically increased the percentage of Pax6+ cells from 6.6% to 31.9%, GLYT1+ cells from 4.0% to 27.4%, calretinin + cells from 3.4% to 31.4%, and TH+ cells from 0.0% to 13.0% (Figure 3Y). Furthermore, smaller increase was observed in the number of GFP+ cells immunoreactive for GABA, Gad65, Gad67 and calbindin in retinas transfected with pCIG-Ptf1a DNA (Figure 3I-N, Q, R, Y). On the other hand, misexpressed Ptf1a decreased the percentage of GFP+ photoreceptor cells immunoreactive for recoverin from 81.8% to 63.5%, GFP+ bipolar cells immunoreactive for Chx10 from 6.7% to 1.1%, and Müller glial cells immunoreactive for GS (glutamine synthetase) from 3.3% to 0.0% (Figure 3S-Y). These data suggest that Ptf1a is able to promote the differentiation of all kinds of amacrine cells including glycinergic, GABAergic and dopaminergic neurons at the expense of photoreceptor, bipolar and Müller cells.

**Figure 4:**
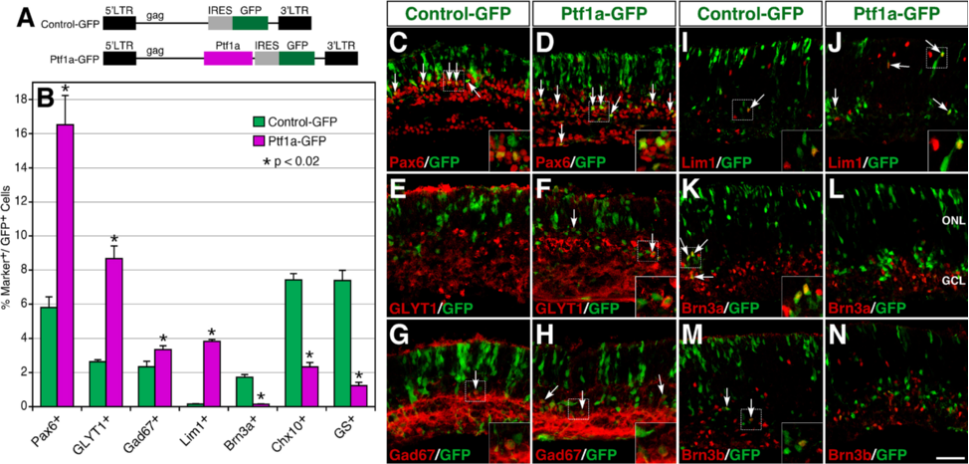
Effect of Ptf1a misexpressed at E13.5 on the formation of different retinal cell types. **(A)** Schematics of control-GFP and Ptf1a-GFP retroviral constructs. The internal ribosomal entry site (IRES) allows for efficient expression of both Ptf1a and GFP. **(B)** Quantitation of GFP+ cells that become immunoreactive for various cell type-specific markers. Each histogram represents the mean ± SD for three retinas. More than 500 GFP+ cells were scored in each retina. **(C-N)** Sections from retinas infected with control-GFP or Ptf1a-GFP viruses were double-immunostained with an anti-GFP antibody and antibodies against the indicated cell type-specific markers. Forced Ptf1a expression in E13.5 retinas led to an increase in amacrine cells immunoreactive for Pax6, GLYT1 or GAD67 **(C-H)** and horizontal cells immunoreactive for Lim1 **(I, J)**, but a decrease of ganglion cells immunoreactive for Brn3a or Brn3b **(K-N)**. Arrows point to representative colocalized cells and insets show corresponding outlined regions at a higher magnification. Abbreviations: GCL, ganglion cell layer; ONL, outer nuclear layer. Scale bar: I-N, 39.7 μm; C-H, 34 μm.

To determine the effect of misexpressed Ptf1a on development of horizontal and ganglion cells, which are born at embryonic stages, we used a replication-incompetent murine retroviral vector that carries a GFP reporter [33] to mediate Ptf1a overexpression. E13.5 retinal explants were infected with Ptf1a-GFP or Control-GFP viruses (Figure 4A), and the infected retinas were harvested after 4.5 days in culture to analyze horizontal and ganglion cells or collected after 12.5 days in culture for analysis of other cell types. We found that misexpressed Ptf1a increased Lim1+ horizontal cells by approximately 25-fold, decreased Brn3a + ganglion cells by 11-fold, and similarly reduced Brn3b + ganglion cells (Figure 4B, I-N). It also significantly increased Pax6+, GLYT1+ and Gad67+ amacrine cells but reduced Chx10+ bipolar and GS+ Müller cells (Figure 4B-H), similar to its effect in retinas transfected at P0 (Figure 3). Therefore, these data suggest that Ptf1a has the ability to not only promote the horizontal cell fate but also suppress the ganglion cell fate, in agreement with the finding in *Ptf1a* null retinas [15,16].

### Tfap2b is expressed in amacrine and horizontal cells and promotes amacrine cell differentiation

**Figure 5:**
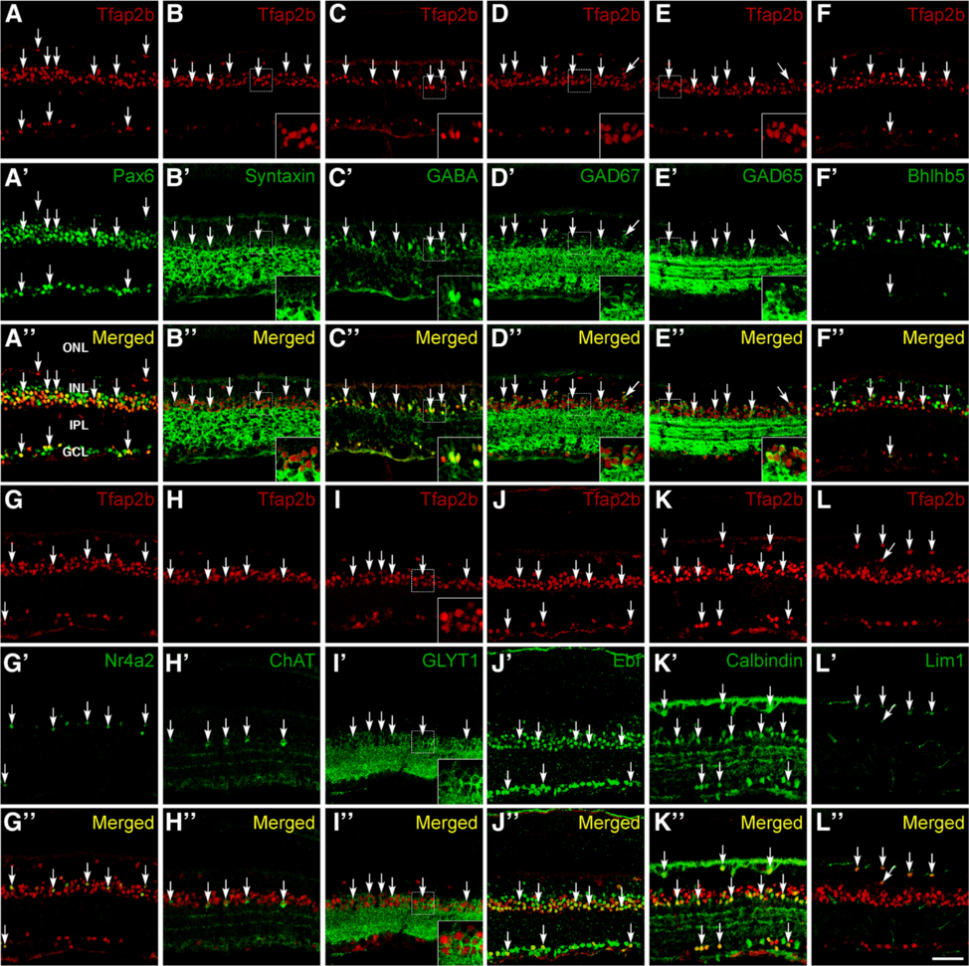
Expression of Tfap2b in amacrine and horizontal cells. **(A-L)** Sections from P21 mouse retinas were double-immunolabeled with an anti-Tfap2b antibody and those against the indicated cell type-specific markers. Tfap2b is expressed in amacrine cells co-expressing Pax6 **(A)**, syntaxin **(B)**, GABA **(C)**, Gad67 **(D)**, Gad65 **(E)**, Bhlhb5 **(F)**, Nr4a2 **(G)**, ChAT **(H)**, GLYT1 **(I)**, Ebf **(J)**, or calbindin **(K)**. It is also expressed by horizontal cells co-expressing calbindin or Lim1 **(K, L)**. However, there is no expression of Tfap2b in Bhlhb5+ bipolar cells within the outer half of the INL **(F)**. Abbreviations: GCL, ganglion cell layer; INL, inner nuclear layer; IPL, inner plexiform layer; ONL, outer nuclear layer. Scale bar: A-L, 47.6 μm.

As a downstream transcription factor, Tfap2b may mediate in part the function of Ptf1a in amacrine and horizontal cell development. To test this possibility, we first comprehensively characterized the types and subtypes of Tfap2b-expressing cells in the mouse retina by immunofluorescence using a battery of cell type- and subtype-specific markers. Consistent with it being expressed in amacrine cells, there is extensive colocalization between Tfap2b and Pax6, syntaxin, GABA, GAD67, GAD65, Nr4a2, ChAT (choline acetyltransferase), GLYT1, Ebf, calbindin, calretinin, or TH (Figure 5A-E, G-K; Additional file 4: Figure S3A, B). Tfap2b appears also to be completely colocalized with calbindin and Lim1 in horizontal cells (Figure 5 K, L; Additional file 4: Figure S3H). There is no expression of Tfap2b in Brn3a + and Brn3b + ganglion cells, Chx10+ bipolar cells, recoverin + photoreceptors, and Sox9+ Müller cells (Additional file 4: Figure S3C-H). Consistent with this, Tfap2b is co-expressed with Bhlhb5 only in a small set of GABAergic amacrine cells since Bhlhb5 is additionally expressed in bipolar cells (Figure 5F; Additional file 4: Figure S3H). Quantification of colocalized amacrine cells revealed that the proportions of Tfap2b-expressing cells in all Pax6+, syntaxin + and GLYT1+ populations are 54.7%, 64.7% and 73.9%, respectively (Additional file 4: Figure S3H), suggesting that Tfap2b is expressed in most but not all glycinergic amacrine cells. However, Tfap2b is expressed in 100% of GABA+, GAD67+, GAD65+, ChAT+, TH+, or Nr4a2+ cells, indicating that it may be expressed by all GABAergic amacrine cells including the starburst (marked by ChAT) and dopaminergic (marked by TH) subtypes.

**Figure 6:**
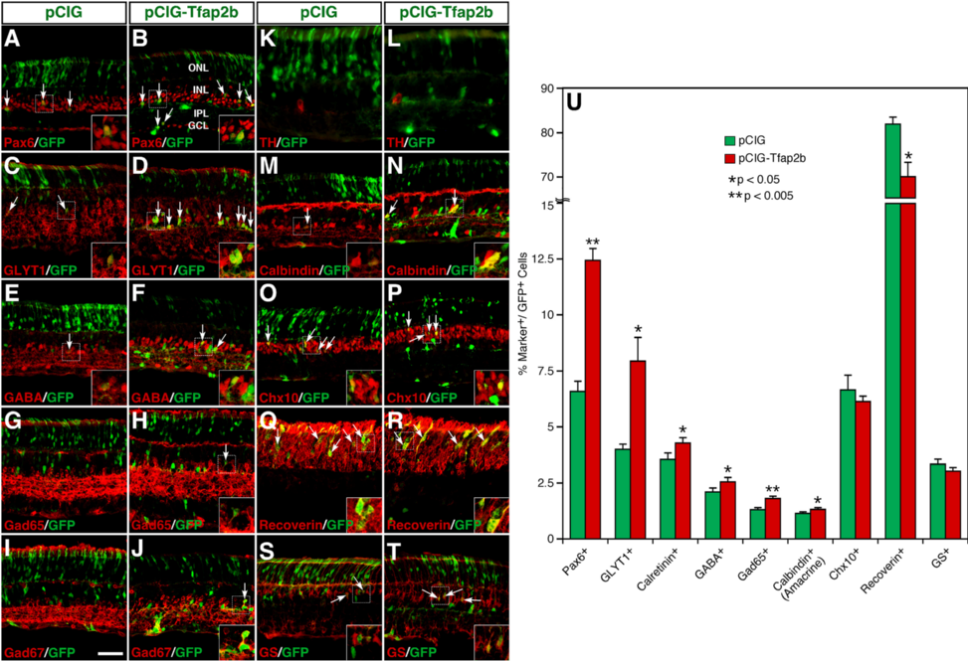
Effect of misexpressed Tfap2b on the differentiation of different retinal cell types. **(A-T)** Sections from retinas electroporated with pCIG or pCIG-Tfap2b DNA were double-immunostained with an anti-GFP antibody and antibodies against the indicated cell type-specific markers. Misexpressed Tfap2b caused a significant increase of amacrine cells immunoreactive for Pax6, GLYT1, GABA, Gad65, Gad67, or calbindin **(A-J, M, N)**, no change in the number of TH-immunoreactive amacrine cells or GS-immunoreactive Müller cells **(K, L, S, T)**, and a significant decrease in the number of recoverin-immunoreactive photoreceptor cells **(Q, R)**. Arrows point to representative colocalized cells and insets show corresponding outlined regions at a higher magnification. **(U)** Quantitation of GFP+ cells that become immunoreactive for various cell type-specific markers. Each histogram represents the mean ± SD for 3–5 retinas. More than 500 GFP+ cells were scored in each retina. Abbreviations: GCL, ganglion cell layer; INL, inner nuclear layer; IPL, inner plexiform layer; ONL, outer nuclear layer. Scale bar: A-J, M-P, S, T, 39.7 μm; K, L, 30.2 μm; Q, R, 23.8 μm.

Tfap2b and 2a are reported to be redundantly required for horizontal cell generation but appear to be dispensable for generating amacrine cells [26,28]. We investigated whether Tfap2b has the ability to promote amacrine cell differentiation by overexpressing it in retinas of newborn mice via electroporation. In P12 transfected retinas, similar to Ptf1a (Additional file 3: Figure S2), we found that overexpressed Tfap2b increased GFP+ cells within the inner half of the INL by 2-fold while significantly diminishing those in the ONL and slightly reducing those in the outer half of the INL (Additional file 5: Figure S4). There was an overt increase in the number of GFP+ cells immunoreactive for Pax6 and GLYT1 in retinas transfected with the pCIG-Tfap2b plasmid compared to the control (Figure 6A-D). Misexpressed Tfap2b increased the fraction of Pax6+ cells from 6.6% to 12.5% and GLYT1+ cells from 4.0% to 8.0% (Figure 6U). It also resulted in smaller but significant increase in the proportion of calretinin+, GABA+, Gad65+, Gad67+ and calbindin + amacrine cells while significantly reducing the fraction of recoverin + photoreceptors (Figure 6E-J, M, N, Q, R, U). However, unlike Ptf1a, misexpressed Tfap2b did not induce the differentiation of TH+ dopaminergic amacrine cells or significantly suppress the differentiation of bipolar and Müller cells (Figure 6 K, L, O, P, U; Figure 3). Thus, Tfap2b has the ability, albeit weaker than that of Ptf1a, to promote the differentiation of both glycinergic and GABAergic amacrine cells.

### Tfap2a facilitates amacrine cell differentiation

As a homolog of Tfap2b, Tfap2a is expected to have a similar role during retinal cell development as Tfap2b. Indeed, we found that Tfap2a misexpressed in retinas of newborn mice significantly increased GFP+ cells distributed within the inner half of the INL and GCL whereas it significantly reduced those in the ONL (Additional file 6: Figure S5A-D). Similar to Tfap2b, it caused obvious increase in the number of GFP+ cells immunoreactive for Pax6 and GLYT1 in transfected retinas (Additional file 6: Figure S5E-H), and smaller increase in the number of GABA+ and Gad65+ GABAergic amacrine cells (Additional file 6: Figure S5I-L), but had no effect on the formation of TH+ dopaminergic neurons (Additional file 6: Figure S5M, N). Thus, Tfap2a may act similarly as Tfap2b to facilitate the differentiation of both glycinergic and GABAergic amacrine cells during retinogenesis.

### Tfap2a and 2b are required for amacrine cell differentiation

To determine whether Tfap2a and 2b are necessary for amacrine cell differentiation, we sought to simultaneously knock down *Tfap2a* and *2b* expression in retinal precursors. To this end, we screened for target sequences (oligonucleotides) in these two genes that are effective in knocking down the expression of *Tfap2a* or *Tfap2b* by shRNA-mediated interference. A Tfap2a shRNA (Tfap2ai5) expressed from the RNAi vector pU6 [34] was found to dramatically reduce GFP expression in HEK293 cells co-transfected with the pCIG-Tfap2a expression plasmid (containing a Tfap2a-IRES-GFP cassette) (Additional file 7: Figure S6A-D). Similarly, we identified a Tfap2b shRNA (Tfap2bi4) that was effective and specific in knocking down *Tfap2b* expression in cell culture (Additional file 7: Figure S6A, E-G).

To investigate whether simultaneous knockdown of both *Tfap2a* and *2b* expression has any functional consequence, we co-electroporated *Tfap2a* and *2b* shRNAs or pU6 plasmid with the pCIG vector into P0 mouse retinas and collected them at P12 for analysis. The proportions of GFP^+^ progeny distributed in different retinal cell layers were quantified. In retinas transfected with both shRNAs (Tfap2ai5 + bi4), compared to the control, the proportion of GFP^+^ cells distributed to the inner half of the INL decreased significantly from 8.9% to 3.3% (Additional file 8: Figure S7); whereas the ratio of GFP^+^ cells differentiated as photoreceptors in the ONL increased from 81.3% to 87.5% (Additional file 8: Figure S7). No change was seen in the proportion of GFP^+^ cells distributed to the outer half of the INL (Additional file 8: Figure S7). The significant reduction of cells in the INL is consistent with the idea that Tfap2a and 2b are redundantly required for proper differentiation of amacrine cells, which normally reside in the inner half of the INL.

**Figure 7:**
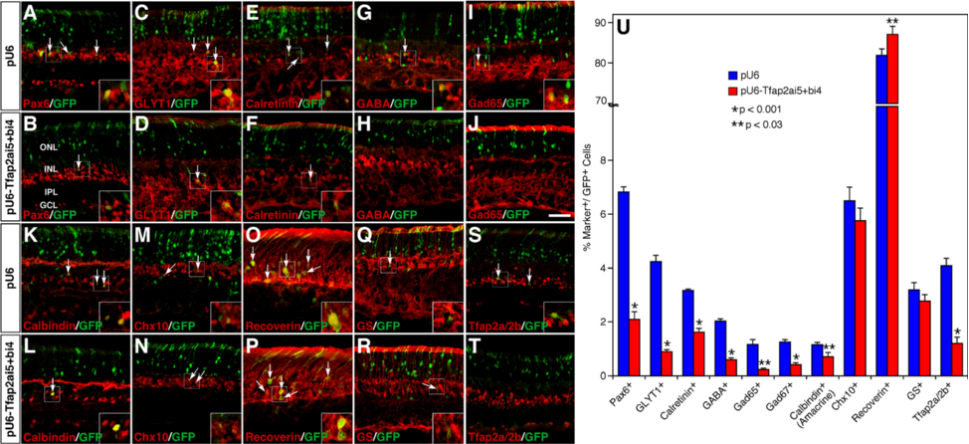
Effect of knocking down *Tfap2a* and *2b* expression on the formation of different retinal cell types. **(A-T)** Sections from retinas co-electroporated with pCIG and RNAi vectors pU6 or pU6-Tfap2ai5 and pU6-Tfap2bi4 were double-immunostained with an anti-GFP antibody and antibodies against the indicated cell type-specific markers. Simultaneous knockdown of both *Tfap2a* and *2b* expression led to a dramatic reduction of Tfap2a- and Tfap2b-immunoreactive cells **(S, T)**, a significant decrease of amacrine cells immunoreactive for Pax6, GLYT1, calretinin, GABA, Gad65, and calbindin **(A-L)**, no change in the number of Chx10-immunoreactive bipolar cells and GS-immunoreactive Müller cells **(M, N, Q, R)**, but a significant increase of recoverin-immunoreactive photoreceptor cells **(O, P)**. Arrows point to representative colocalized cells and insets show corresponding outlined regions at a higher magnification. **(U)** Quantitation of GPF+ cells that are immunoreactive for various cell type-specific markers. Each histogram represents the mean ± SD for 3 retinas. More than 600 GFP+ cells were scored in each retina. Abbreviations: GCL, ganglion cell layer; INL, inner nuclear layer; IPL, inner plexiform layer; ONL, outer nuclear layer. Scale bar: A-N, Q-T, 39.7 μm; O, P, 23.8 μm.

Transfection of *Tfap2a* and *2b* shRNAs led to more than 3-fold decrease in the fraction of GFP+ cells immunolabeled by the antibody cross-reacting with both Tfap2a and 2b (Figure 7S-U), demonstrating the effectiveness of the double knockdown strategy. Consistent with decreased GFP+ cells in the INL, it caused a significant reduction of amacrine cells immunostained by various molecular markers including Pax6, GLYT1, calretinin, GABA, GAD65, GAD67, and calbindin (Figure 7A-L, U). For instance, in retinas transfected with both shRNAs, the proportion of Pax6+, GLYT1+, GABA+ and GAD65+ cells decreased from 6.8%, 4.2%, 2.0% and 1.2% to 2.1%, 0.9%, 0.6% and 0.2%, respectively (Figure 7U). By contrast, misexpression of *Tfap2a* and *2b* shRNAs increased the percentage of recoverin + photoreceptors from 81.9% to 87.1% and had no effect on the differentiation of Ch10+ bipolar cells and GS+ Müller cells (Figure 7 M-R, U). These results thus indicate that Tfap2a and 2b factors are not only sufficient but also necessary for promoting the differentiation of glycinergic and GABAergic amacrine cells during retinal development.

## Discussion

### Ptf1a acts as a general regulator to specify different amacrine and horizontal cell subtypes

Ptf1a is required to specify GABAergic versus glutamatergic neurons in the mouse spinal cord and cerebellum [35,36]. In the *Xenopus* retina, it also preferentially promotes the formation of GABAergic amacrine and horizontal cells [19]. However, in the mouse retina, Ptf1a appears to be necessary for specifying all amacrine and horizontal cell types [15,16]. In the chick retina, its overexpression promotes all horizontal cell subtypes including GABAergic H1/H2 cells as well as non-GABAergic H3 cells [18]. Similarly, in the zebrafish retina, Ptf1a is expressed by all subtypes of amacrine cells and appears to be necessary and sufficient for their specification [20,37]. In this work, we show that in the mouse retina, Ptf1a has the ability to promote the fates of not only GABAergic but also glycinergic amacrine cells, and in fact, it has a much more potent activity to promote glycinergic than GABAergic amacrine neuron differentiation (Figure 3). Therefore, our data lend support to the idea that Ptf1a may act as a general transcriptional regulator to specify all subtypes of amacrine and horizontal cells during retinal development, although its function may exhibit some species specificity.

### Tfap2a and 2b function downstream of Ptf1a for amacrine and horizontal cell differentiation

Given the essential role for Ptf1a in specifying subsets of neurons in the CNS, efforts have been undertaken to identify its downstream targets during neural development [38-40]. For instance, *Neurog2* has been found to act as a direct Ptf1a target in the specification of GABAergic neurons in the dorsal spinal cord and cerebellum, and *Nephrin* and *Neph3* expression is directly regulated by Ptf1a in developing neurons [39,40]. However, the downstream genes of Ptf1a involved in retinal development are yet to be identified. In this study, we profiled transcriptomes of wild type and *Ptf1a* mutant retinas by RNA-seq and identified *Tfap2a* and *2b* as two transcription factor genes prominently downregulated in the mutant retina. Interestingly, our previous microarray profiling analysis has identified the same two genes as those significantly downregulated in *Foxn4* mutant retinas [29]. Given the known epistatic relationship between Foxn4 and Ptf1a during retinal development [15,17], however, *Tfap2a* and *2b* are unlikely to be direct targets of Foxn4.

Previous studies have shown that both Tfap2a and 2b are essential for embryonic development and involved in eye morphogenesis. Targeted deletion of either gene in mice causes perinatal lethality [41-43]. *Tfap2a* inactivation results in anencephaly, craniofacial cleft, thoraco-abdominoschisis, and lens defect [42-44]. Similarly, mutations in human *TFAP2A* are associated with the Branchio-Oculo-Facial Syndrome with variable ocular anomalies including microphthalmia or anophthalmia, iris and chorioretinal coloboma, strabismus, and cataract [24,25,45,46]. During mouse retinogenesis, Tfap2a and 2b are overlappingly expressed in postmitotic amacrine and horizontal cells [26-28]. Conditional inactivation of *Tfap2a* fails to cause any retinal defect [26]. However, when the conditional *Tfap2a* line was bred with the *Tfap2b* conventional knockout strain to obtain double knockout embryos, there was a complete loss of horizontal cells, indicating that Tfap2a and 2b are redundantly required for horizontal cell differentiation [28], consistent with the expected role for them as Ptf1a downstream effectors. On the other hand, there was no obvious change in the number of amacrine cells in the double mutant retina except for a minor migratory defect [28], begging the question whether Tfap2a and 2b can mediate the function of Ptf1a in amacrine cell differentiation.

We utilized both overexpression and knockdown approaches to assess the role of Tfap2a and 2b in amacrine cell development. Previous studies have shown that many of the transcription factors involved in retinal development act as both positive and negative regulators depending on the cell types. For example, Barhl2 promotes glycinergic amacrine cell differentiation while negatively regulating the formation of bipolar and Müller cells [33]. Rax1 and Hes1 are able to promote the Müller glial cell fate as well as inhibit neuronal cell differentiation [47]. Similarly, we are able to show that misexpressed Tfap2a and 2b can function as positive factors to promote differentiation of glycinergic and GABAergic amacrine cells while negatively regulating the formation of photoreceptors. Therefore, Tfap2a and 2b indeed can fulfill their expected role in mediating the function of Ptf1a in amacrine cell differentiation. However, it appears that they have a much weaker activity than Ptf1a in facilitating and suppressing retinal cell types: 1) the number of glycinergic amacrine neurons induced by misexpressed Tfap2b is about 3-fold less than those induced by Ptf1a (compare Figures 3, 6); 2) neither Tfap2a nor 2b is able to promote the formation of TH+ dopaminergic amacrine cells while Ptf1a has a potent activity to do so; and 3) Tfap2b is unable to inhibit bipolar and Müller cell differentiation while Ptf1a is. Thus, Tfap2a and 2b are able to mediate only part of the Ptf1a function in amacrine cell development and there should be other downstream factors that participate as Ptf1a effectors.

To investigate the necessity for Tfap2a and 2b in amacrine cell differentiation, we simultaneously knocked down *Tfap2a* and *2b* expression by RNAi in newborn retinal precursors to circumvent the issue of functional redundancy. This genetic manipulation resulted in a significant reduction of glycinergic and GABAergic amacrine cells accompanied with a concomitant increase of photoreceptors. This result tallies well with the gain-of-function data to demonstrate that Tfap2a and 2b are both necessary and sufficient to promote amacrine cell differentiation, but differs from that of the reported *Tfap2a* and *2b* double mutant retina where no obvious amacrine cell loss was seen [28]. This discrepancy may result from the fact that further analysis of amacrine cell differentiation was impossible beyond the neonatal stage due to perinatal lethality of the double mutant embryo on the conventional *Tfap2b* knockout background [28]. It would be necessary to achieve retina-specific inactivation of both *Tfap2a* and *2b* to circumvent the lethal phenotype and resolve the discrepancy.

### Transcriptional regulatory pathways to amacrine and horizontal cell development

**Figure 8:**
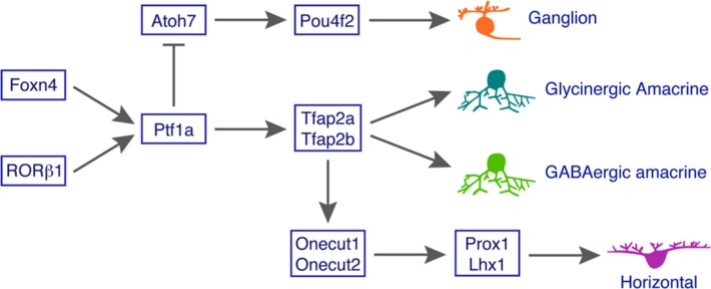
Model by which Tfap2 and Ptf1a factors promote the differentiation of amacrine and horizontal cells. Tfap2a and 2b act downstream of Ptf1a to promote the differentiation of glycinergic and GABAergic amacrine cells while functioning upstream of Onecut1 and 2 for the specification of horizontal cells. Ptf1a, whose expression is activated by both Foxn4 and ROR 1, is required not only for specifying amacrine and horizontal cells in part by activating *Tfap2a* and *2b* expression, but also for suppressing the ganglion cell fate by repressing *Atoh7* expression.

Despite the requirement of Pax6 for retinal progenitors to acquire multipotency, the absence of *Pax6* in mice permits the formation of amacrine cells [48], implicating that other factors are sufficient to make the progenitors competent for the generation of amacrine cells. The identity of these additional regulators have been gradually revealed over time. Targeted inactivation of *Foxn4*, *ROR*β*1 or Ptf1a* in mice result in a similar retinal phenotype, i.e. elimination of horizontal cells and loss of the great majority of amacrine cells, whereas their misexpression in newborn retinas leads to increased amacrine cells (Figure 3) [13,15,16,21]. The absence of either *Foxn4* or *ROR*β*1* causes marked downregulation of *Ptf1a* expression but *Foxn4* expression remains unchanged in the *Ptf1a* mutant retina [15,21,29]. Our RNA-seq data also reveal no significant change in the expression level of *Rorb* in the *Ptf1a* mutant. Given the normal expression of *Foxn4* in the *ROR*β*1* null retina [21], Foxn4 and RORβ1 are likely to act in parallel upstream of Ptf1a (Figure 8), and indeed are shown to directly and synergistically activate *Ptf1a* expression through binding to enhancer elements [21].

Through combined bioinformatic and genetic analyses, we are able to show that *Tfap2a* and *2b* are dramatically downregulated in the *Ptf1a* null retina, and their knockdown causes decreased amacrine cell differentiation whereas their overexpression leads to the opposite outcome. Given that knockout of both *Tfap2a* and *2b* results in essentially complete loss of horizontal cells [28], we propose a Foxn4/RORβ1-Ptf1a-Tfap2a/2b transcriptional regulatory cascade that underlies the competence, specification and differentiation of amacrine and horizontal cells during retinal development (Figure 8). Onecut1 and Onecut2 have been shown to be required for horizontal cell differentiation by activating the expression of *Prox1* and *Lhx1*, two transcription factor genes also involved in horizontal cell development [49-52]. However, they appear not to be required for activating *Tfap2a* and *2b* expression because RNA-seq reveals that there is no change of *Tfap2a* expression and even significant upregulation of *2b* expression in *Onecut1* and *2* double mutant retinas [52]. Since *Onecut1* and *2* expression is downregulated in *Tfap2a* and *2b* double mutant retinas (Judith West-Mays, personal communication), these two Tfap2 factors are likely to function upstream of the Onecut factors to control horizontal cell differentiation (Figure 8). We and others have also shown that *Ptf1a* inactivation causes overproduction of retinal ganglion cells while its misexpression inhibits ganglion cell differentiation and expression of *Atoh7* (Figure 4) [15,16,18], a transcription factor essential for conferring the ganglion cell competence [53-55]. Thus, Ptf1a appears to ensure proper specification of competent precursors to amacrine and horizontal cells not only by promoting these two cell fates but also by suppressing the alternative ganglion cell fate (Figure 8).

## Methods

### Mice

All procedures in animals were performed according to the IACUC standards, and approved by Rutgers, the State University of New Jersey, and/or by Zhongshan Ophthalmic Center and Sun Yat-Sen University. All mice were maintained and bred in the university vivarium with normal diet. The C57BL6/J mice were obtained from the Jackson Laboratory (Bar Harbor, ME). The CD1 mice were purchased from the Charles River Laboratories (Wilmington, MA). The *Foxn4* and *Ptf1a* knockout mouse lines were reported previously [13,56] and maintained by breeding with C57BL6/J mice. The starting stage of mouse embryos was defined as E0.5 when the copulation plug was seen in the morning. Mouse genotype was determined by standard PCR.

### RNA-Seq

Total RNA was extracted from E14.5 wild type and *Ptf1a* null mutant retinas using the TRIzol reagent (Invitrogen) according to the manufacturer’s instruction. Ribosomal RNA was depleted prior to RNA-seq library preparation. The prepared DNA was sequenced using an Illumina HiSeq 2000 sequencer. The obtained sequence reads were trimmed and mapped to the mouse reference genome (mm10) using Tophat [57] (http://ccb.jhu.edu/software/tophat/index.shtml). Gene expression and changes were analyzed using Cufflinks (http://cole-trapnell-lab.github.io/cufflinks/), whose output was then further analyzed by CummeRbund (http://compbio.mit.edu/cummeRbund/) and iReport (Qiagen). Cluster analysis was performed using Genesis [58] and GO term enrichment using DAVID [59] (http://david.abcc.ncifcrf.gov/).

### Antibodies and immunostaining

Tissue processing and immunostaining were carried out as described previously [13,32]. The following primary antibodies were used: mouse anti-Brn3a (1:100, Cat: MAB1585, Millipore); goat anti-Brn3b (1:200, Cat:sc-6026, Santa Cruz Biotech.); goat anti-Bhlhb5/BETA3 (1:800, Cat: sc-6045, Santa Cruz Biotech.); rabbit anti-calbindin D-28 k (1:3000, Cat: CB-38, Swant); mouse anti-calretinin (1:1000, Cat: mab1568, Millipore); goat anti-choline acetyltransferase (ChAT) (1:300, Cat: AB144P, Millipore); sheep anti-Chx10 (1:1600, Cat: x1180p, Exalpha); rabbit anti-Dab1 (1:200, Cat: sc-13981, Santa Cruz Biotech.); goat anti-EBF (1:40, Cat:sc-15888, Santa Cruz Biotech.); rabbit anti-GABA (1:3000, Cat: a2052, Sigma); mouse anti-Gad65 (1:5000, Cat: 559931, BD Biosciences); mouse anti-Gad67 (1:1000, Cat: mab5406, Millipore); rabbit anti-GFP (1:800, Cat: 598, MBL International); Goat anti-GFP (1:1500, Cat: ab6673, Abcam); mouse anti-glutamine synthetase (1:5000, Cat: mab302, Millipore); goat anti-GLYT1 (1:4000, Cat: AB1770, Millipore); mouse anti-Lim1/2 [1:100, Cat: 4 F2, Developmental Studies Hybridoma Bank (DSHB)]; rabbit anti-Nr4a2/Nurr1 (1:1000, Cat: sc-990, Santa Cruz Biotech.); rabbit anti-Pax6 (1:2000, Cat: ab5409, Millipore); rabbit anti-recoverin (1:10000, Cat: ab5585, Millipore); Mouse anti-syntaxin (1:3000, Cat: S0664, Sigma); rabbit anti-Sox9 (1:1000, Cat: ab5535, Millipore); rabbit anti-Tfap2a/2b (1:500, Cat: sc-184, Santa Cruz Biotech.); rabbit anti-Tfap2b (1:100, Cat: sc-8976, Santa Cruz Biotech.); mouse anti-Tfap2a (1:200, Cat: 5E4, DSHB); and rabbit anti-tyrosine hydroxylase (1:2000, Cat: ab152, Millipore). Secondary antibodies conjugated with fluorophore Alexa 488 or 594 were purchased from Life Technologies. Images were captured by a Leica TCS-SP2 confocal system or with a Nikon Eclipse 80i microscope.

### Plasmid electroporation and virus infection

The pCIG vector was reported previously [30-32]. It is a mammalian expression vector containing the CMV enhancer, chicken β-actin promoter, multiple cloning sites (MCS), IRES-eGFP and rabbit β-globin PolyA sequences. For misexpression experiments, the full-length ORFs of mouse Ptf1a, Tfap2a and Tfap2b were amplified from retinal cDNA and subcloned into the MCS of the pCIG vector. 0.5-2 μg of each plasmid was injected into the subretinal space at P0 and electroporated into retinal cells [60]. Injected retinas were collected at P12 for analysis.

The methods to prepare retroviruses, infect retinas and collect samples are described in detail previously [32,60]. For retrovirus preparation, the full-length ORF of mouse Ptf1a was cloned into the MMLV-based replication-incomplete retroviral vector pLZRSΔ-IRES-GFP [33]. E13.5 retinal explants were infected with retroviruses and harvested after 4.5 days in culture to analyze horizontal and ganglion cells or collected after 12.5 days in culture for analysis of other cell types.

### shRNA plasmids and RNAi interference

For RNAi knockdown experiments, selected small hairpin sequences were inserted into the shRNA interference vector pBS/U6 containing the human U6 promoter [34]. Knockdown efficiency was tested by cotransfection into 293 cells of the shRNA plasmid, pmCherry-N1 vector and pCIG construct containing the corresponding gene. To rule out differences in transfection efficiency, pmCherry-N1 was used as a red fluorescence marker to monitor transfection efficiency. The constructs with the best knockdown efficiency were used in the retinal knockdown experiments. The targeting sequence used for Tfap2a is: 5’-AACATTCCGATCCCAATGAGC-3’ (Tfap2ai5), and for Tfap2b is: 5’-CTACTCAGTTCAACTTCAAAGTACA-3’ (Tfap2bi4). To perform retinal knockdown, pBS/U6 constructs and pCIG vector (as a GFP reporter) were mixed at a ratio of 2:1 (μg/μl) and 1 μl of the mixture was injected into the subretinal space of P0 CD1 mice. Electroporation was carried out immediately following injection [32]. Transfected retinas were collected at P12 when the great majority of retinal cells are determined and developed into mature cell types.

### Semi-quantitative RT-PCR

Total RNA was isolated from E14.5 wild type and *Ptf1a* mutant mouse retinas, and cDNA was made using the NEB Reverse Transcription Kit. The following primers were used for semi-quantitative RT-PCR (all from 5’ to 3’): *Tfap2a*, GCCTGAATCCTCTGCACGC and GTCCTCGTGCCGCCGATA; *Tfap2b*, CCAGCTCTCCGGCCTTGATC and CAACTGACTGCACGTCTTCCATG; *Gapdh*, CGTGCCGCCTGGAGAAACCTG and GAGTGGGAGTTGCTGTTGAAGTCGC. The products were amplified for 27 cycles and visualized on a 1.5% agarose gel.

### Quantification and statistical analysis

For misexpression experiments, depending on the frequency or ratio of each cell type, hundreds to thousands of GFP+ cells in each infected retina were scored; at least 3 retinas were used for each individual cell marker. All quantification data were subjected to significance test using two sample Student’s t-test with unequal variances and two tails.

## Additional files

Additional file 1: Table S1. List of genes differentially expressed between E14.5 wild type and Ptf1a mutant retinas. Additional file 2: Figure S1. Expression of Tfap2b during mouse retinal development. (A-D) Retinal sections from the indicated developmental stages were immunostained with an anti-Tfap2b antibody and weakly counterstained with DAPI. Tfap2b-immunoreactive cells are seen in scattered cells at E13.5 in the central retina, and gradually become concentrated in the presumptive inner nuclear layer from E14.5 to E16.5 (B-D). (**E-G)** A P21 retinal section was double-immunolabeled with anti-Tfap2a and anti-Tfap2b antibodies. Tfap2a and 2b are colocalized in the great majority of immunoreactive cells. Arrows point to labeled horizontal cells. Abbreviations: GCL, ganglion cell layer; INL, inner nuclear layer; IPL, inner plexiform layer; L, lens; ONBL, outer neuroblastic layer; ONL, outer nuclear layer; OPL, outer plexiform layer; R, retina. Scale bar in C: A, B, 50 μm; C, D, 25 μm. Scale bar in G: E-G, 47.6 μm. Additional file 3: Figure S2. Ptf1a misexpression alters the distribution pattern and morphology of retinal cells. (A) Schematics of the pCIG and pCIG-Ptf1a expression plasmids. The internal ribosomal entry site (IRES) allows for efficient expression of both Ptf1a and GFP. (**B, C**) Transfected GFP+ cells were visualized in retinal sections that were weakly counterstained with TOPRO3. Ptf1a misexpression causes an obvious increase of GFP+ cells located in the INL but a reduction of photoreceptors residing in the ONL. (**D**) Percentages of GFP+ cells located in different cellular layers of the retina (means ± SD). Three retinas were scored for each virus and more than 700 GFP^+^ cells were counted in each retina. Abbreviations: GCL, ganglion cell layer; INL, inner nuclear layer; IPL, inner plexiform layer; ONL, outer nuclear layer; and OPL, outer plexiform layer. Scale bar: B, C, 39.7 μm. Additional file 4: Figure S3. Expression of Tfap2b in retinal cell types. (A-G) Sections from P21 mouse retinas were double-immunolabeled with an anti-Tfap2b antibody and those against the indicated cell type-specific protein markers. Tfap2b is colocalized with calretinin and TH in amacrine cells, but not expressed in Brn3a- or Brn3b-immunoreactive ganglion cells, Chx10-immunoreactive bipolar cells, Sox9-immunoreactive Müller cells, or in recoverin-immunoreactive photoreceptors. (**H**) Percentages of marker-positive retinal cells that are immunoreactive for Tfap2b. Abbreviations: GCL, ganglion cell layer; INL, inner nuclear layer; IPL, inner plexiform layer; ONL, outer nuclear layer. Scale bar: A-G, 47.6 μm. Additional file 5: Figure S4. Tfap2b misexpression alters the distribution pattern and morphology of retinal cells. (A) Schematics of the pCIG and pCIG-Tfap2b expression plasmids. (**B, C**) Transfected GFP+ cells were visualized in retinal sections that were weakly counterstained with TOPRO3. Tfap2b misexpression causes an increase of GFP+ cells located in the INL but a decrease of photoreceptors residing in the ONL. (**D**) Percentages of GFP+ cells located in different cellular layers of the retina (means ± SD). Three retinas were scored for each virus and more than 700 GFP^+^ cells were counted in each retina. Abbreviations: GCL, ganglion cell layer; INL, inner nuclear layer; IPL, inner plexiform layer; ONL, outer nuclear layer; and OPL, outer plexiform layer. Scale bar: B, C, 39.7 μm. Additional file 6: Figure S5. Effect of misexpressed Tfap2a on the differentiation of different retinal cell types. (A) Schematics of the pCIG and pCIG-Tfap2a expression plasmids. (**B**, **C**) Transfected GFP+ cells were visualized in retinal sections that were weakly counterstained with DAPI. Tfap2a misexpression results in an increase of GFP+ cells located in the INL but a decrease of photoreceptors residing in the ONL. (**D**) Percentages of GFP+ cells located in different cellular layers of the retina (means ± SD). Three retinas were scored for each virus and more than 1000 GFP^+^ cells were counted in each retina. (**E-N)** Sections from retinas electroporated with pCIG or pCIG-Tfap2a DNA were double-immunostained with an anti-GFP antibody and antibodies against the indicated cell type-specific markers. Misexpressed Tfap2a increased amacrine cells immunoreactive for Pax6, GLYT1, GABA, or Gad65 (E-L), but not the number of TH-immunoreactive dopaminergic neurons (M, N). Arrows point to representative colocalized cells and insets show corresponding outlined regions at a higher magnification. Abbreviations: GCL, ganglion cell layer; INL, inner nuclear layer; IPL, inner plexiform layer; ONL, outer nuclear layer; and OPL, outer plexiform layer. Scale bar: B, C, E-L, 39.7 μm; M, N, 30.2 μm. Additional file 7: Figure S6. Specificity of the *Tfap2a* and *2b* shRNA. (A) Schematics of Tfap2a and 2b protein structural domains and regions targeted by the corresponding shRNA. AD, activation domain; HSH, helix-span-helix motif. (**B-G**) In transfected HEK293 cells, the Tfap2ai5 shRNA greatly reduced expression of the Tfap2a-IRES-GFP cassette, as marked by GFP; whereas it had no effect on the expression of Tfap2b (B,C,E,F). The opposite was true for the Tfap2bi4 shRNA (B,D,E,G). Comparable transfection efficiency was observed by the presence of similar number of mCherry-expressing cells co-transfected with the pmCherry-N1 expression plasmid (B’-G’). Additional file 8: Figure S7. Reducing *Tfap2a* and *2b* expression alters the distribution pattern of retinal cells. (A, B) Simultaneous transfection of *Tfap2a* and *2b* shRNA caused a significant decrease of GFP+ cells distributed in the INL. (**C**) Percentages of GFP+ cells located in different cellular layers of retinas transfected with pU6 or with both pU6-Tfap2ai5 and pU6-Tfap2bi4 plasmids (mean ± SD). Three retinas were scored for each plasmid and more than 900 GFP^+^ cells were counted in each retina. Abbreviations: GCL, ganglion cell layer; INL, inner nuclear layer; IPL, inner plexiform layer; ONL, outer nuclear layer; and OPL, outer plexiform layer. Scale bar: A, B, 39.7 μm.

## Abbreviations

ChAT: Choline acetyltransferase
GABA: γ-aminobutyric acid
GAD: Glutamic acid decarboxylase
GFP: Green fluorescent protein
GLYT1: Glycine transporter 1
GO: Gene ontology
GS: Glutamine synthetase
INL: Inner nuclear layer
IRES: Internal ribosome entry site
MMLV: Moloney murine leukemia virus
ONL: Outer nuclear layer
ORF: Open reading frame
shRNA: Short hairpin ribonucleic acid
TH: Tyrosine hydroxylase

## References

[1] Livesey FJ, Cepko CL (2001). Vertebrate neural cell-fate determination: lessons from the retina. Nat Rev Neurosci.

[2] Harris WA (1997). Cellular diversification in the vertebrate retina. Curr Opin Genet Dev.

[3] Yang XJ (2004). Roles of cell-extrinsic growth factors in vertebrate eye pattern formation and retinogenesis. Semin Cell Dev Biol.

[4] Xiang M (2013). Intrinsic control of mammalian retinogenesis. Cell Mol Life Sci.

[5] Masland RH (2001). The fundamental plan of the retina. Nat Neurosci.

[6] Masland RH (2001). Neuronal diversity in the retina. Curr Opin Neurobiol.

[7] MacNeil MA, Heussy JK, Dacheux RF, Raviola E, Masland RH (1999). The shapes and numbers of amacrine cells: matching of photofilled with Golgi-stained cells in the rabbit retina and comparison with other mammalian species. J Comp Neurol.

[8] MacNeil MA, Masland RH (1998). Extreme diversity among amacrine cells: implications for function. Neuron.

[9] Menger N, Pow DV, Wassle H (1998). Glycinergic amacrine cells of the rat retina. J Comp Neurol.

[10] Pourcho RG, Goebel DJ (1985). A combined Golgi and autoradiographic study of (^3^H)glycine-accumulating amacrine cells in the cat retina. J Comp Neurol.

[11] Vaney DI (1990). The mosaic of amacrine cells in the mammalian retina. Prog Retinal Res.

[12] Pourcho RG, Goebel DJ (1983). Neuronal subpopulations in cat retina which accumulate the GABA agonist, (^3^H)muscimol: a combined Golgi and autoradiographic study. J Comp Neurol.

[13] Li S, Mo Z, Yang X, Price SM, Shen MM, Xiang M (2004). Foxn4 controls the genesis of amacrine and horizontal cells by retinal progenitors. Neuron.

[14] Boije H, Shirazi Fard S, Ring H, Hallbook F (2013). Forkheadbox N4 (FoxN4) triggers context-dependent differentiation in the developing chick retina and neural tube. Differentiation.

[15] Fujitani Y, Fujitani S, Luo H, Qiu F, Burlison J, Long Q (2006). Ptf1a determines horizontal and amacrine cell fates during mouse retinal development. Development.

[16] Nakhai H, Sel S, Favor J, Mendoza-Torres L, Paulsen F, Duncker GI (2007). Ptf1a is essential for the differentiation of GABAergic and glycinergic amacrine cells and horizontal cells in the mouse retina. Development.

[17] Xiang M, Li S (2013). Foxn4: a multi-faceted transcriptional regulator of cell fates in vertebrate development. Sci China Life Sci.

[18] Lelievre EC, Lek M, Boije H, Houille-Vernes L, Brajeul V, Slembrouck A (2011). Ptf1a/Rbpj complex inhibits ganglion cell fate and drives the specification of all horizontal cell subtypes in the chick retina. Dev Biol.

[19] Dullin JP, Locker M, Robach M, Henningfeld KA, Parain K, Afelik S (2007). Ptf1a triggers GABAergic neuronal cell fates in the retina. BMC Dev Biol.

[20] Jusuf PR, Almeida AD, Randlett O, Joubin K, Poggi L, Harris WA (2011). Origin and determination of inhibitory cell lineages in the vertebrate retina. J Neurosci.

[21] Liu H, Kim SY, Fu Y, Wu X, Ng L, Swaroop A (2013). An isoform of retinoid-related orphan receptor β directs differentiation of retinal amacrine and horizontal interneurons. Nat Commun.

[22] Eckert D, Buhl S, Weber S, Jager R, Schorle H (2005). The AP-2 family of transcription factors. Genome Biol.

[23] Hilger-Eversheim K, Moser M, Schorle H, Buettner R (2000). Regulatory roles of AP-2 transcription factors in vertebrate development, apoptosis and cell-cycle control. Gene.

[24] Milunsky JM, Maher TA, Zhao G, Roberts AE, Stalker HJ, Zori RT (2008). *TFAP2A* mutations result in branchio-oculo-facial syndrome. Am J Hum Genet.

[25] Milunsky JM, Maher TM, Zhao G, Wang Z, Mulliken JB, Chitayat D (2011). Genotype-phenotype analysis of the branchio-oculo-facial syndrome. Am J Med Genet A.

[26] Bassett EA, Pontoriero GF, Feng W, Marquardt T, Fini ME, Williams T (2007). Conditional deletion of activating protein 2α (AP-2α) in the developing retina demonstrates non-cell-autonomous roles for AP-2α in optic cup development. Mol Cell Biol.

[27] Bassett EA, Williams T, Zacharias AL, Gage PJ, Fuhrmann S, West-Mays JA (2010). AP-2α knockout mice exhibit optic cup patterning defects and failure of optic stalk morphogenesis. Hum Mol Genet.

[28] Bassett EA, Korol A, Deschamps PA, Buettner R, Wallace VA, Williams T (2012). Overlapping expression patterns and redundant roles for AP-2 transcription factors in the developing mammalian retina. Dev Dyn.

[29] Luo H, Jin K, Xie Z, Qiu F, Li S, Zou M (2012). Forkhead box N4 (Foxn4) activates Dll4-Notch signaling to suppress photoreceptor cell fates of early retinal progenitors. Proc Natl Acad Sci U S A.

[30] Megason SG, McMahon AP (2002). A mitogen gradient of dorsal midline Wnts organizes growth in the CNS. Development.

[31] Misra K, Luo H, Li S, Matise M, Xiang M (2014). Asymmetric activation of Dll4-Notch signaling by Foxn4 and proneural factors activates BMP/TGFβ signaling to specify V2b interneurons in the spinal cord. Development.

[32] Jin K, Jiang H, Mo Z, Xiang M (2010). Early B-cell factors are required for specifying multiple retinal cell types and subtypes from postmitotic precursors. J Neurosci.

[33] Mo Z, Li S, Yang X, Xiang M (2004). Role of the *Barhl2* homeobox gene in the specification of glycinergic amacrine cells. Development.

[34] Sui G, Soohoo C, Affar el B, Gay F, Shi Y, Forrester WC (2002). A DNA vector-based RNAi technology to suppress gene expression in mammalian cells. Proc Natl Acad Sci U S A.

[35] Glasgow SM, Henke RM, Macdonald RJ, Wright CV, Johnson JE (2005). Ptf1a determines GABAergic over glutamatergic neuronal cell fate in the spinal cord dorsal horn. Development.

[36] Hoshino M, Nakamura S, Mori K, Kawauchi T, Terao M, Nishimura YV (2005). *Ptf1a*, a bHLH transcriptional gene, defines GABAergic neuronal fates in cerebellum. Neuron.

[37] Jusuf PR, Harris WA (2009). Ptf1a is expressed transiently in all types of amacrine cells in the embryonic zebrafish retina. Neural Dev.

[38] Wildner H, Das Gupta R, Brohl D, Heppenstall PA, Zeilhofer HU, Birchmeier C (2013). Genome-wide expression analysis of *Ptf1a*- and *Ascl1*-deficient mice reveals new markers for distinct dorsal horn interneuron populations contributing to nociceptive reflex plasticity. J Neurosci.

[39] Nishida K, Hoshino M, Kawaguchi Y, Murakami F (2010). Ptf1a directly controls expression of immunoglobulin superfamily molecules Nephrin and Neph3 in the developing central nervous system. J Biol Chem.

[40] Henke RM, Savage TK, Meredith DM, Glasgow SM, Hori K, Dumas J (2009). Neurog2 is a direct downstream target of the Ptf1a-Rbpj transcription complex in dorsal spinal cord. Development.

[41] Moser M, Pscherer A, Roth C, Becker J, Mucher G, Zerres K (1997). Enhanced apoptotic cell death of renal epithelial cells in mice lacking transcription factor AP-2β. Genes Dev.

[42] Schorle H, Meier P, Buchert M, Jaenisch R, Mitchell PJ (1996). Transcription factor AP-2 essential for cranial closure and craniofacial development. Nature.

[43] Zhang J, Hagopian-Donaldson S, Serbedzija G, Elsemore J, Plehn-Dujowich D, McMahon AP (1996). Neural tube, skeletal and body wall defects in mice lacking transcription factor AP-2. Nature.

[44] West-Mays JA, Zhang J, Nottoli T, Hagopian-Donaldson S, Libby D, Strissel KJ (1999). AP-2α transcription factor is required for early morphogenesis of the lens vesicle. Dev Biol.

[45] Aliferis K, Stoetzel C, Pelletier V, Helle S, Angioi-Duprez K, Vigneron J (2011). A novel *TFAP2A* mutation in familial Branchio-Oculo-Facial Syndrome with predominant ocular phenotype. Ophthalmic Genet.

[46] Dumitrescu AV, Milunsky JM, Longmuir SQ, Drack AV (2012). A family with branchio-oculo-facial syndrome with primarily ocular involvement associated with mutation of the *TFAP2A* gene. Ophthalmic Genet.

[47] Furukawa T, Mukherjee S, Bao ZZ, Morrow EM, Cepko CL (2000). *rax*, *Hes1*, and *notch1* promote the formation of Müller glia by postnatal retinal progenitor cells. Neuron.

[48] Marquardt T, Ashery-Padan R, Andrejewski N, Scardigli R, Guillemot F, Gruss P (2001). Pax6 is required for the multipotent state of retinal progenitor cells. Cell.

[49] Wu F, Li R, Umino Y, Kaczynski TJ, Sapkota D, Li S (2013). Onecut1 is essential for horizontal cell genesis and retinal integrity. J Neurosci.

[50] Poche RA, Kwan KM, Raven MA, Furuta Y, Reese BE, Behringer RR (2007). Lim1 is essential for the correct laminar positioning of retinal horizontal cells. J Neurosci.

[51] Dyer MA, Livesey FJ, Cepko CL, Oliver G (2003). Prox1 function controls progenitor cell proliferation and horizontal cell genesis in the mammalian retina. Nat Genet.

[52] Sapkota D, Chintala H, Wu F, Fliesler SJ, Hu Z, Mu X: Onecut1 and Onecut2 redundantly regulate early retinal cell fates during development. Proc Natl Acad Sci U S A. 2014;111:E4086–95. 10.1073/pnas.1405354111 PMC4191802 25228773

[53] Brown NL, Patel S, Brzezinski J, Glaser T (2001). Math5 is required for retinal ganglion cell and optic nerve formation. Development.

[54] Wang SW, Kim BS, Ding K, Wang H, Sun D, Johnson RL (2001). Requirement for *math5* in the development of retinal ganglion cells. Genes Dev.

[55] Yang Z, Ding K, Pan L, Deng M, Gan L (2003). Math5 determines the competence state of retinal ganglion cell progenitors. Dev Biol.

[56] Kawaguchi Y, Cooper B, Gannon M, Ray M, MacDonald RJ, Wright CV (2002). The role of the transcriptional regulator Ptf1a in converting intestinal to pancreatic progenitors. Nat Genet.

[57] Trapnell C, Roberts A, Goff L, Pertea G, Kim D, Kelley DR (2012). Differential gene and transcript expression analysis of RNA-seq experiments with TopHat and Cufflinks. Nat Protoc.

[58] Sturn A, Quackenbush J, Trajanoski Z (2002). Genesis: cluster analysis of microarray data. Bioinformatics.

[59] da Huang W, Sherman BT, Lempicki RA (2009). Systematic and integrative analysis of large gene lists using DAVID bioinformatics resources. Nat Protoc.

[60] Jin K, Xiang M (2012). In vitro explant culture and related protocols for the study of mouse retinal development. Methods Mol Biol.

