# Supplementary figures and images for: Tfap2a and 2b act downstream of Ptf1a to promote amacrine cell differentiation during retinogenesis
Source: Mol Brain. 2015 May 13;8:28. doi: 10.1186/s13041-015-0118-x (PMC4429372; doi:10.1186/s13041-015-0118-x)

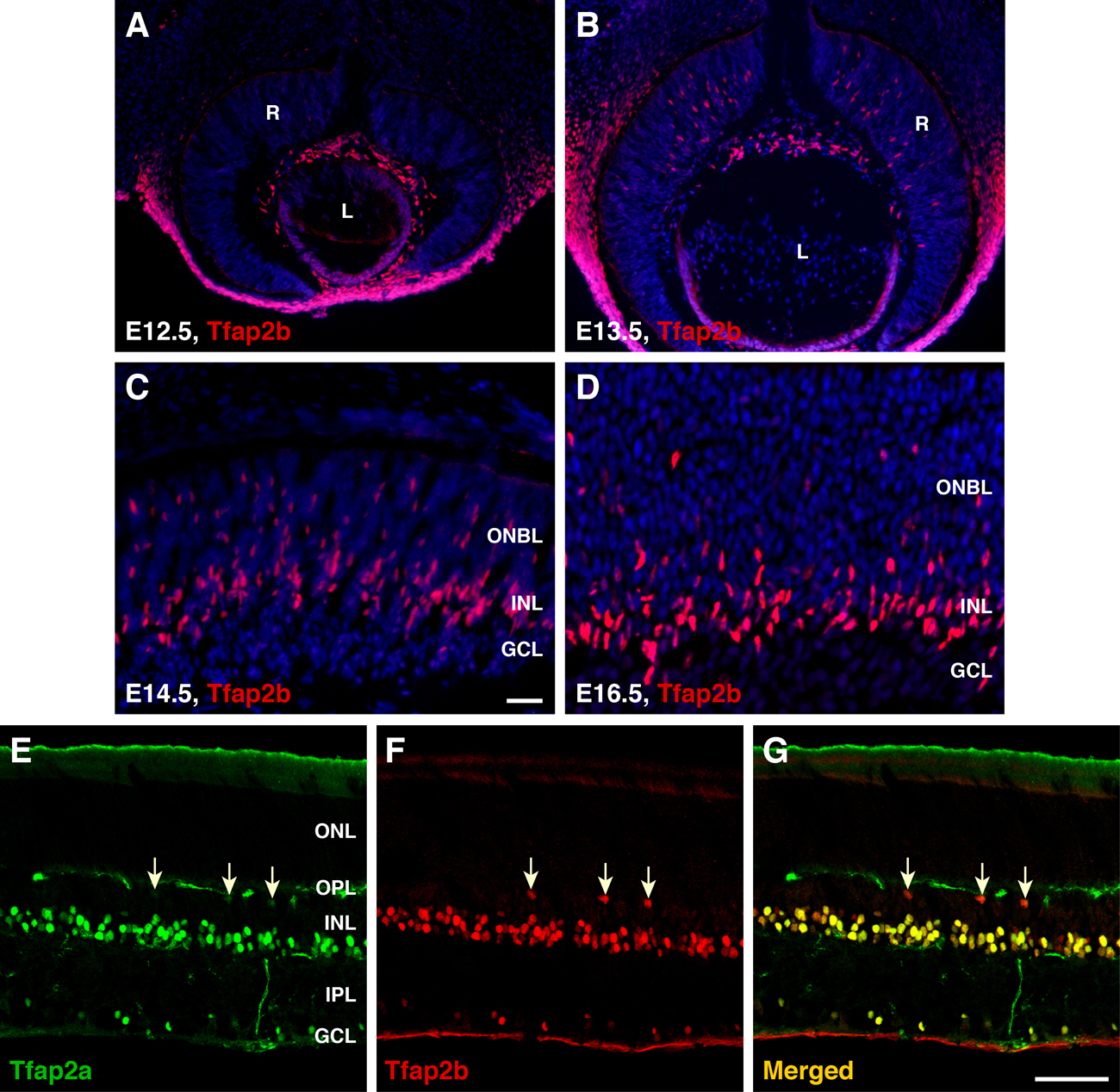

Supplement: Additional file 2: Figure S1. — Expression of Tfap2b during mouse retinal development. (A-D) Retinal sections from the indicated developmental stages were immunostained with an anti-Tfap2b antibody and weakly counterstained with DAPI. Tfap2b-immunoreactive cells are seen in scattered cells at E13.5 in the central retina, and gradually become concentrated in the presumptive inner nuclear layer from E14.5 to E16.5 (B-D). (E-G) A P21 retinal section was double-immunolabeled with anti-Tfap2a and anti-Tfap2b antibodies. Tfap2a and 2b are colocalized in the great majority of immunoreactive cells. Arrows point to labeled horizontal cells. Abbreviations: GCL, ganglion cell layer; INL, inner nuclear layer; IPL, inner plexiform layer; L, lens; ONBL, outer neuroblastic layer; ONL, outer nuclear layer; OPL, outer plexiform layer; R, retina. Scale bar in C: A, B, 50 μm; C, D, 25 μm. Scale bar in G: E-G, 47.6 μm. [file 13041_2015_118_MOESM2_ESM.jpeg]

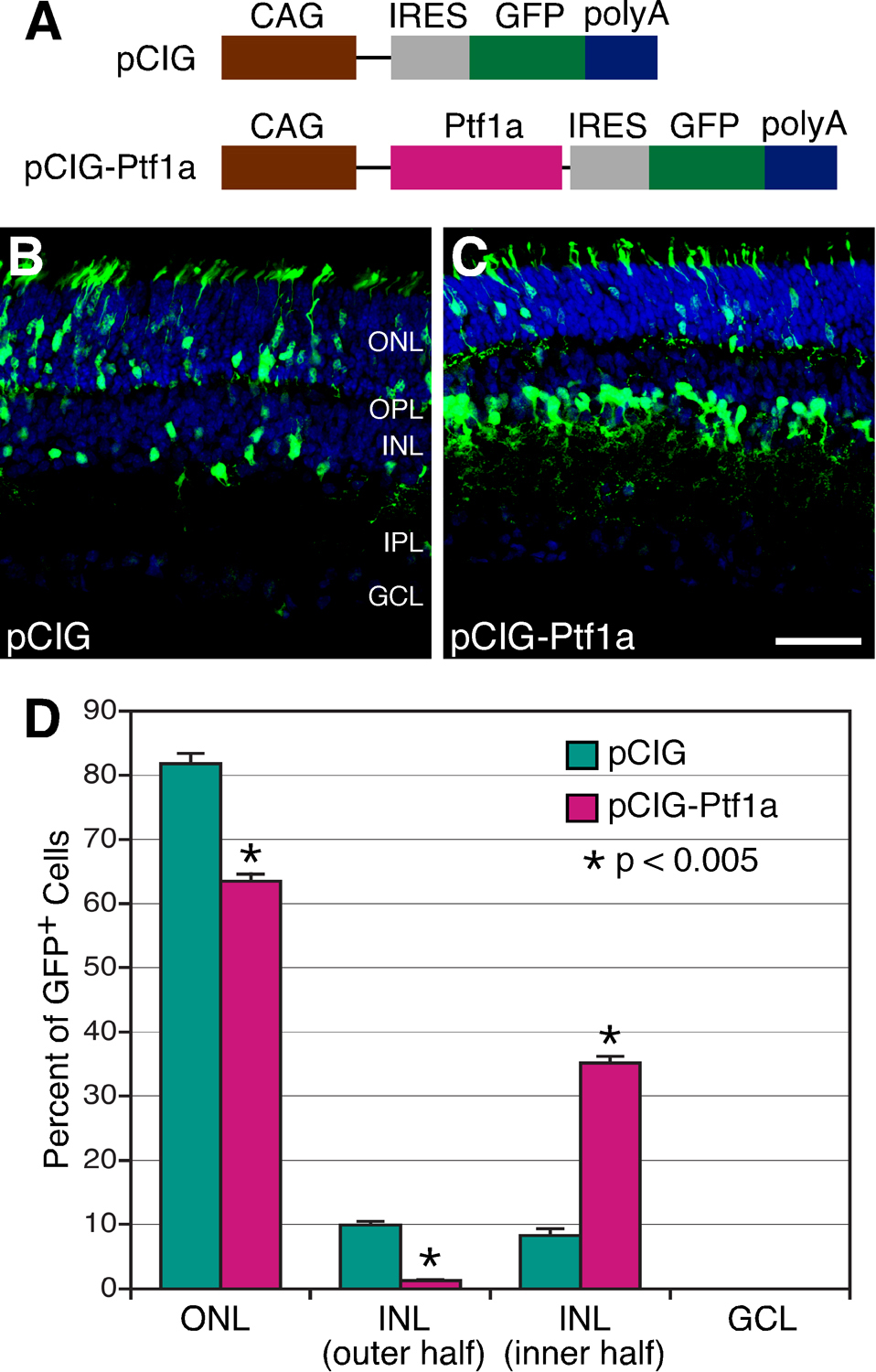

Supplement: Additional file 3: Figure S2. — Ptf1a misexpression alters the distribution pattern and morphology of retinal cells. (A) Schematics of the pCIG and pCIG-Ptf1a expression plasmids. The internal ribosomal entry site (IRES) allows for efficient expression of both Ptf1a and GFP. (B, C) Transfected GFP+ cells were visualized in retinal sections that were weakly counterstained with TOPRO3. Ptf1a misexpression causes an obvious increase of GFP+ cells located in the INL but a reduction of photoreceptors residing in the ONL. (D) Percentages of GFP+ cells located in different cellular layers of the retina (means ± SD). Three retinas were scored for each virus and more than 700 GFP+ cells were counted in each retina. Abbreviations: GCL, ganglion cell layer; INL, inner nuclear layer; IPL, inner plexiform layer; ONL, outer nuclear layer; and OPL, outer plexiform layer. Scale bar: B, C, 39.7 μm. [file 13041_2015_118_MOESM3_ESM.jpeg]

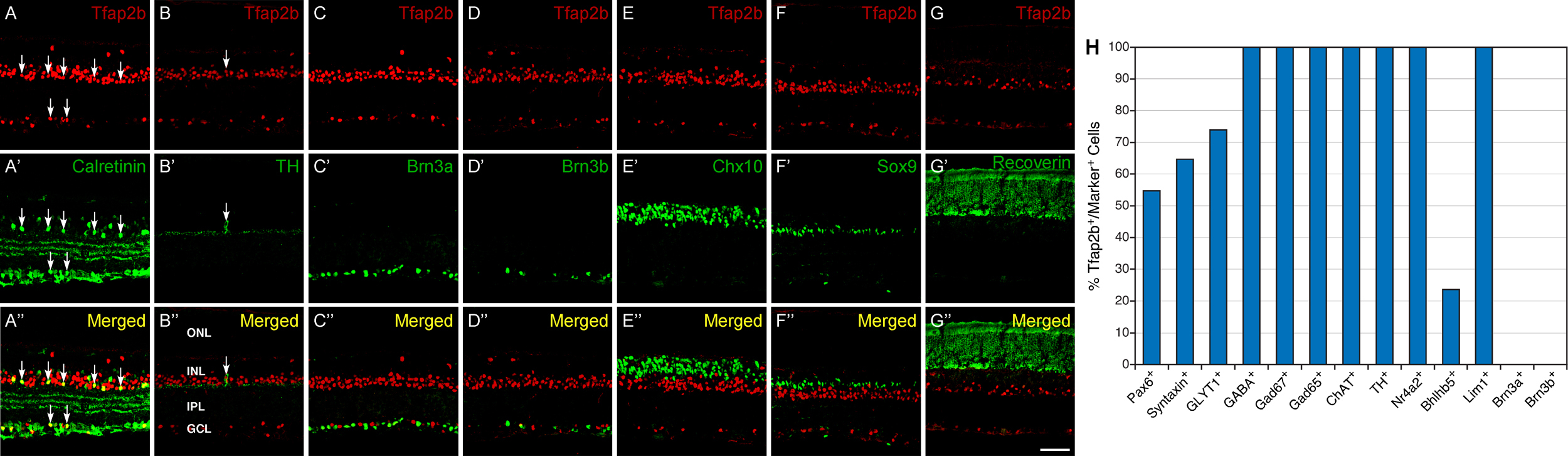

Supplement: Additional file 4: Figure S3. — Expression of Tfap2b in retinal cell types. (A-G) Sections from P21 mouse retinas were double-immunolabeled with an anti-Tfap2b antibody and those against the indicated cell type-specific protein markers. Tfap2b is colocalized with calretinin and TH in amacrine cells, but not expressed in Brn3a- or Brn3b-immunoreactive ganglion cells, Chx10-immunoreactive bipolar cells, Sox9-immunoreactive Müller cells, or in recoverin-immunoreactive photoreceptors. (H) Percentages of marker-positive retinal cells that are immunoreactive for Tfap2b. Abbreviations: GCL, ganglion cell layer; INL, inner nuclear layer; IPL, inner plexiform layer; ONL, outer nuclear layer. Scale bar: A-G, 47.6 μm. [file 13041_2015_118_MOESM4_ESM.jpeg]

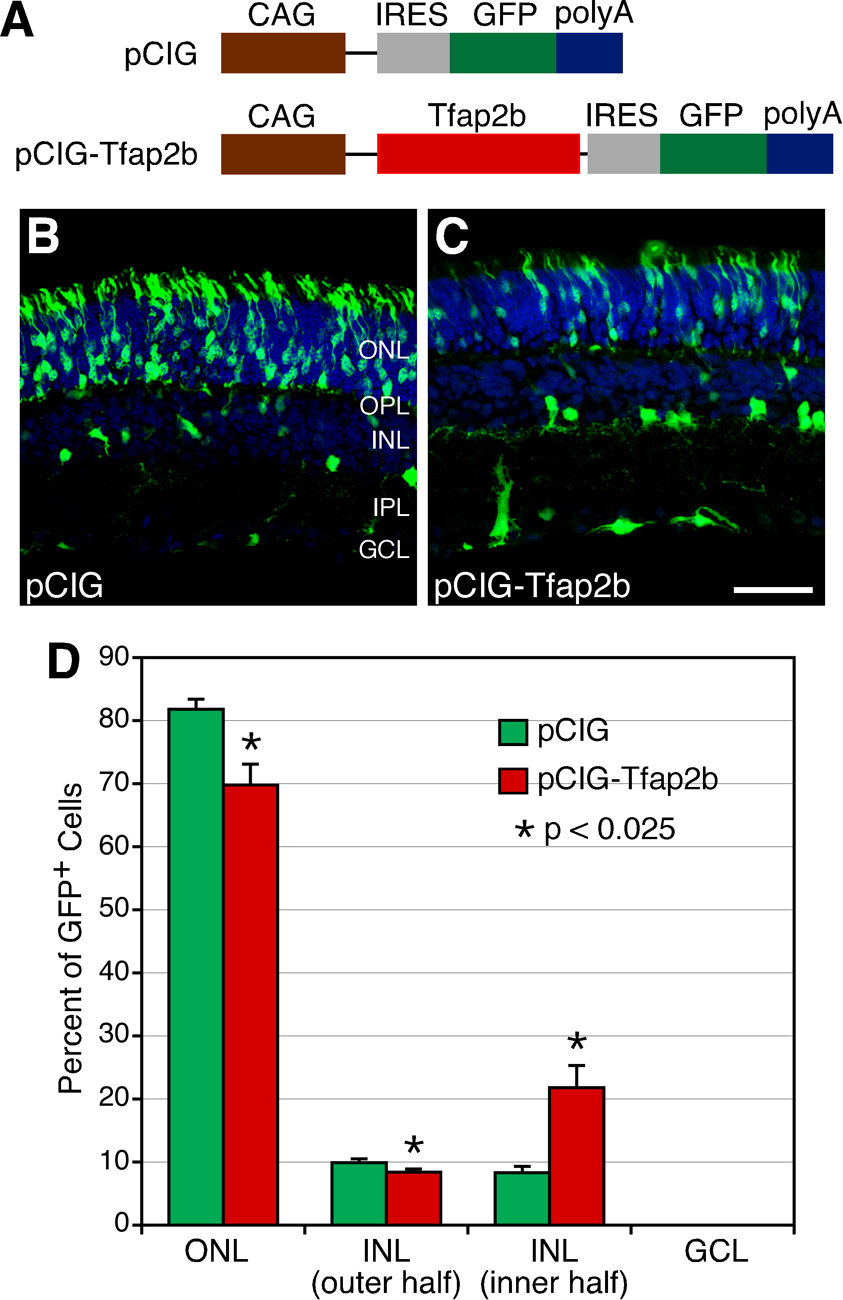

Supplement: Additional file 5: Figure S4. — Tfap2b misexpression alters the distribution pattern and morphology of retinal cells. (A) Schematics of the pCIG and pCIG-Tfap2b expression plasmids. (B, C) Transfected GFP+ cells were visualized in retinal sections that were weakly counterstained with TOPRO3. Tfap2b misexpression causes an increase of GFP+ cells located in the INL but a decrease of photoreceptors residing in the ONL. (D) Percentages of GFP+ cells located in different cellular layers of the retina (means ± SD). Three retinas were scored for each virus and more than 700 GFP+ cells were counted in each retina. Abbreviations: GCL, ganglion cell layer; INL, inner nuclear layer; IPL, inner plexiform layer; ONL, outer nuclear layer; and OPL, outer plexiform layer. Scale bar: B, C, 39.7 μm. [file 13041_2015_118_MOESM5_ESM.jpeg]

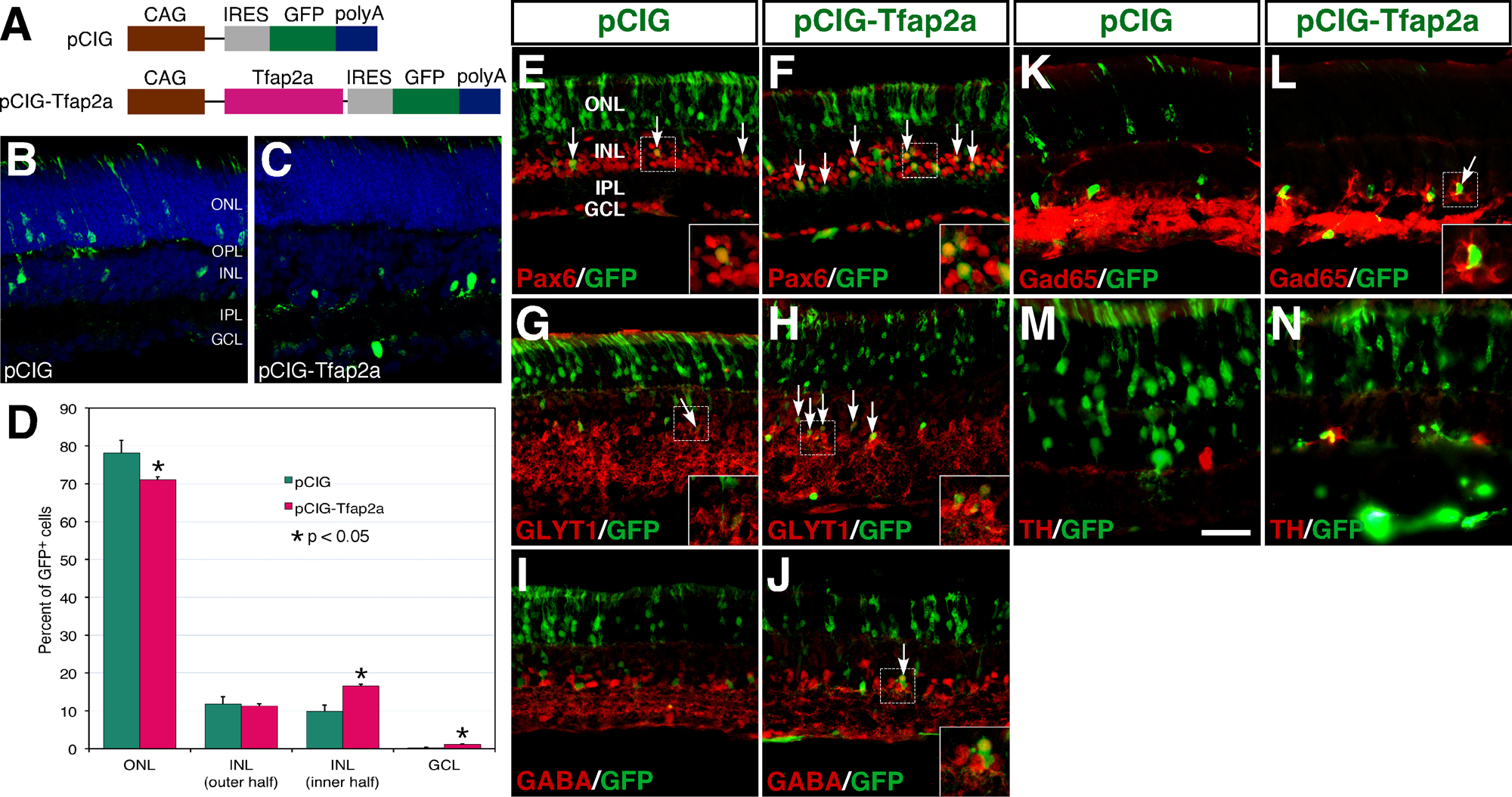

Supplement: Additional file 6: Figure S5. — Effect of misexpressed Tfap2a on the differentiation of different retinal cell types. (A) Schematics of the pCIG and pCIG-Tfap2a expression plasmids. (B, C) Transfected GFP+ cells were visualized in retinal sections that were weakly counterstained with DAPI. Tfap2a misexpression results in an increase of GFP+ cells located in the INL but a decrease of photoreceptors residing in the ONL. (D) Percentages of GFP+ cells located in different cellular layers of the retina (means ± SD). Three retinas were scored for each virus and more than 1000 GFP+ cells were counted in each retina. (E-N) Sections from retinas electroporated with pCIG or pCIG-Tfap2a DNA were double-immunostained with an anti-GFP antibody and antibodies against the indicated cell type-specific markers. Misexpressed Tfap2a increased amacrine cells immunoreactive for Pax6, GLYT1, GABA, or Gad65 (E-L), but not the number of TH-immunoreactive dopaminergic neurons (M, N). Arrows point to representative colocalized cells and insets show corresponding outlined regions at a higher magnification. Abbreviations: GCL, ganglion cell layer; INL, inner nuclear layer; IPL, inner plexiform layer; ONL, outer nuclear layer; and OPL, outer plexiform layer. Scale bar: B, C, E-L, 39.7 μm; M, N, 30.2 μm. [file 13041_2015_118_MOESM6_ESM.jpeg]

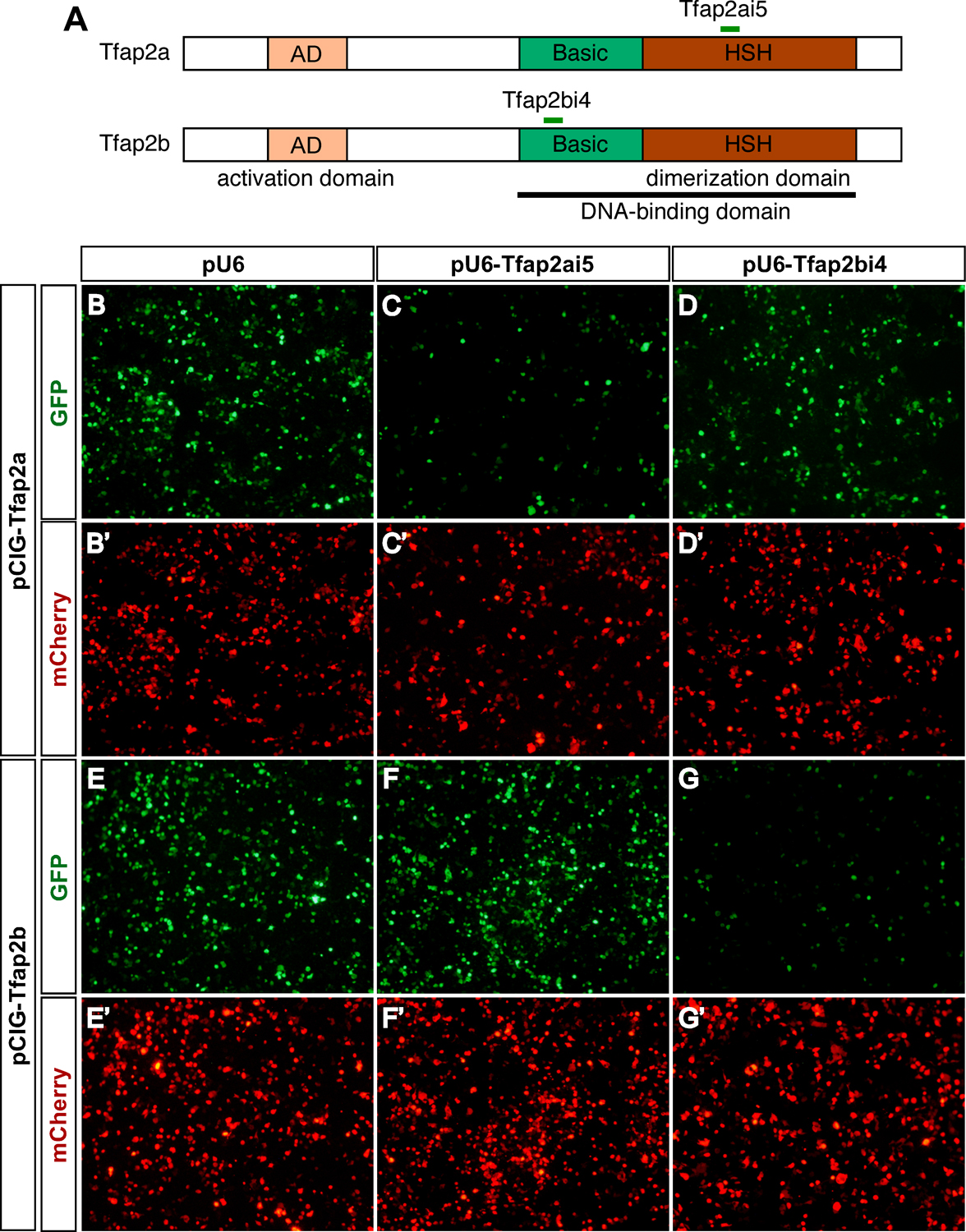

Supplement: Additional file 7: Figure S6. — Specificity of the Tfap2a and 2b shRNA. (A) Schematics of Tfap2a and 2b protein structural domains and regions targeted by the corresponding shRNA. AD, activation domain; HSH, helix-span-helix motif. (B-G) In transfected HEK293 cells, the Tfap2ai5 shRNA greatly reduced expression of the Tfap2a-IRES-GFP cassette, as marked by GFP; whereas it had no effect on the expression of Tfap2b (B,C,E,F). The opposite was true for the Tfap2bi4 shRNA (B,D,E,G). Comparable transfection efficiency was observed by the presence of similar number of mCherry-expressing cells co-transfected with the pmCherry-N1 expression plasmid (B’-G’). [file 13041_2015_118_MOESM7_ESM.jpeg]

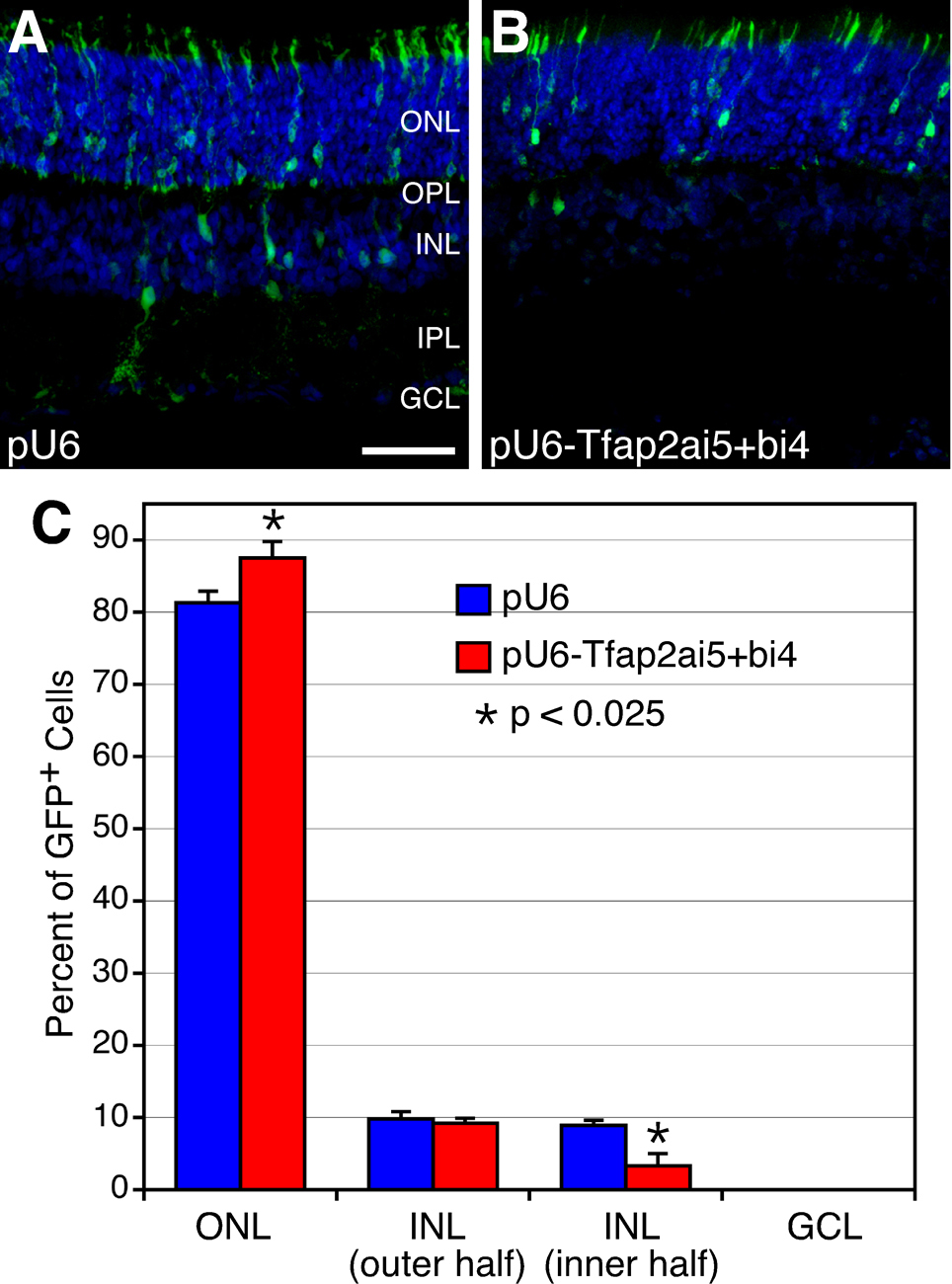

Supplement: Additional file 8: Figure S7. — Reducing Tfap2a and 2b expression alters the distribution pattern of retinal cells. (A, B) Simultaneous transfection of Tfap2a and 2b shRNA caused a significant decrease of GFP+ cells distributed in the INL. (C) Percentages of GFP+ cells located in different cellular layers of retinas transfected with pU6 or with both pU6-Tfap2ai5 and pU6-Tfap2bi4 plasmids (mean ± SD). Three retinas were scored for each plasmid and more than 900 GFP+ cells were counted in each retina. Abbreviations: GCL, ganglion cell layer; INL, inner nuclear layer; IPL, inner plexiform layer; ONL, outer nuclear layer; and OPL, outer plexiform layer. Scale bar: A, B, 39.7 μm. [file 13041_2015_118_MOESM8_ESM.jpeg]
